# Risk Assessment of Heavy Metals in Soils from Four Different Industrial Plants in a Medium-Sized City in North China

**DOI:** 10.3390/toxics11030217

**Published:** 2023-02-25

**Authors:** Dejun Yang, Huawei Zhu, Jianqin Liu, Yajun Zhang, Song Wu, Jibing Xiong, Fayuan Wang

**Affiliations:** 1 Engineering Research Center of Ministry of Education for Mine Ecological Restoration, China University of Mining and Technology, Xuzhou 221116, China; 2 School of Environment Science and Spatial Informatics, China University of Mining and Technology, Xuzhou 221116, China; 3 Jiangsu Fangzheng Environmental Protection Group Co., Ltd., Xuzhou 221006, China; 4 College of Environment and Safety Engineering, Qingdao University of Science and Technology, Qingdao 266042, China

**Keywords:** carcinogenic effect, Nemerow pollution index, single pollution index, spatial distribution

## Abstract

Laboratory experiments were carried out to analyze 39 soil samples collected from four industrial areas in Xuzhou City using inductively coupled plasma mass spectrometry and atomic fluorescence spectrometry. The descriptive statistics of heavy metals (HMs) in the soil profiles showed that the HM content at three depths was highly variable, and most coefficients of variation (CVs) showed moderate variability. The enrichment of Cd at all depths exceeded the risk screening value, and Cd pollution occurred in four plants. The enrichment of the other HMs at three depths was mainly concentrated in the pharmaceutical plant A and chemical plant C. It was found that the different HMs had different vertical distribution characteristics. For the different industrial plants, the raw materials and products not only made the spatial distribution characteristics of the HMs different, but also caused the HM types and contents to differ. The average single pollution indices of Cd in plant A, iron-steel plant B, and plant C indicated a slight pollution level. The other seven HMs in A, B, and C and all HMs in chemical plant D belonged to the safe category. The mean values of the Nemerow pollution index in the four industrial plants belonged to the warning category. The analysis showed that none of the HMs posed potential noncarcinogenic health risks, and only the carcinogenic health risks of Cr in plants A and C were unacceptable. The carcinogenic effect of Cr through the inhalation intake of resuspended soil particulates and that of Cd, Ni, and As via direct oral ingestion were the main exposure pathways.

## 1. Introduction

Generally, heavy metals (HMs) refer to copper (Cu), lead (Pb), zinc (Zn), nickel (Ni), chromium (Cr), cadmium (Cd), and mercury (Hg). Metalloid arsenic (As) also belongs to the HM category, due to its similarity of chemical properties and environmental behaviors [1]. With rapid urbanization and industrialization, the soil environment has been polluted, especially by HMs [2,3]. The soil pollution induced by HMs is considered a globally challenging issue [4].

Soil HM pollution has been widespread and serious across China since the economic reform and opening in the late 1970s [5,6]. Some statistics show that over 10 million hectares of land in China are threatened by soil HM pollution [7]. According to an extensive survey on the environmental quality of soils from 2005 to 2013 by the Ministry of Environmental Protection and the Ministry of Land and Resources [8], it was shown that 16.1% of sampling sites exceeded the environmental quality standards in agricultural soils, and HMs accounted for 82.4% of contaminated samples [9]. Statistically, 7.0%, 1.6%, 2.7%, 2.1%, 1.5%, 1.1%, 0.9%, and 4.8% of soils in China exceeded the limit for Cd, Hg, As, Cu, Pb, Cr, Zn, and Ni, respectively [9].

In recent years, soil HM pollution has attracted social attention and raised widespread concern regarding the ecological environment, public health, and food safety. The Chinese government has implemented many policies to definitively address the soil environment, including the Technical Guidelines for Environmental Site Investigation, Technical Guidelines for Environmental Site Monitoring [10], Technical Guidelines of Soil and Groundwater Self-Monitoring for Enterprises in Production (Draft for Comments), and Soil Environmental Quality: Risk Control Standard for Soil Contamination of Agricultural Land [11]. Furthermore, the Soil Pollution Prevention and Control Law of the People’s Republic of China was published on 31 August 2018 and has been effective since 1 January 2019.

HMs in soils exhibit a nonbiodegradable and persistent nature throughout agricultural, urban, and industrial lands [12]; having a long-term impact on soil physical, chemical, and biological properties, and leading to many environmental problems [13,14]. At the same time, soil physical, chemical, and biological indicators, such as soil parent material, temperature, pH, the degree of oxidation and reduction, and microorganisms, can also directly or indirectly affect the background content of HMs. The absorption of plants and their adsorption and cooperation with other charged ions can affect the severity of environmental problems [15]. HMs can be absorbed by the human body through inhalation, ingestion, and dermal absorption [14,16,17,18]. The food chain is another non-negligible route by which HMs threaten human health. Although some HMs, such as Cu and Zn, are required for the normal growth and functioning of living organisms [17], the accumulation of HMs can have a harmful influence on human health. For example, long-term exposure to carcinogenic HMs (Cd, Cr, As, and Pb) can raise cancer risks [19,20]. Carcinogenic health risk is usually used to estimate the cancer risk caused by an individual’s exposure to carcinogens through three pathways during their lifetime.

Numerous investigations on soil HM pollution have been carried out in agricultural regions, such as contaminated paddy fields in Xiangtan [21]; farmland in Tai’an [22] and Huainan [23]; and roadside agricultural soils of Amritsar and the Tarn Taran district in Punjab, India [24]; as well as in urban regions, such as parks and green areas in Seville [25]; the urban–rural transition zone in Changchun [26]; several functional areas [27] and residential areas in Beijing [28]; and the Porto urban area [29]. Soil HM pollution status has also been investigated in industrial regions, such as the industrial area of Surat [30], the industrial areas of Uttar Pradesh [31], a replaced urban industrial area in Qingdao [32], and four villages around non-ferrous mining and smelting sites [33].

HMs in farmland are mainly introduced from industrial activities, sewage irrigation, and the overuse of pesticides and fertilizers [34,35], while those in industrial lands originate from industrial processes, including pharmaceutical, smelting, dyeing, and other chemical processes [25]. Many studies have proven that industrial activities have a negative impact on the soil environment and that different specific metals might be linked to different industrial activities. For example, Pb, Ni, Cu, and Co are often used as catalysts, modifiers, and dryers [36]. Cr, Zn, Pb, and Ni are linked to tannery activity, agrochemical production (fertilizer), oil refinery activity, and petrochemical emissions, respectively [36,37].

Many studies have been conducted on pollution levels, health risks, and source apportionment of soil HMs [38,39]. Different HMs in soils show differences in mobility and availability, resulting in different vertical and spatial distribution characteristics. For example, the solubility of Cd can increase in acidic soil, resulting in a stronger migration and enrichment ability [40]. In comparison, Pb’s mobility in soil is poor, partly because it is easily adsorbed by organic matter or minerals after entering the soil. The assessment of the environmental health risks can provide basic presumptions for the identification of remediation measurements for reducing the negative impacts on human health and food safety [16]. Differences between industrial areas, industrial plants, and chemical processes may influence the conclusions drawn in different studies. However, few health risk assessment studies of soil HM pollution have been focused on different industrial plants. Studies on the spatial distribution characteristics of HMs in soils and the assessment of their environmental health risks are helpful for understanding the sources of soil HM pollution and making decisions regarding soil remediation in industrial plants.

Most studies related to industrial soil HM pollution were carried out in large cities, and less information is available for the medium-sized cities in China. At present, the medium-sized cities in China, which have relatively poor environmental management systems, are facing increased environmental problems, due to rapid urbanization and industrialization. The environmental problems induced in other medium-sized cities around the world are similar to those in China.

Xuzhou is a typical medium-sized city in North China, with a gross domestic product of 675.523 billion RMB in 2018 and a permanent population of 8.802 million at the end of 2018. To our knowledge, there has been little information published on the pollution level, spatial distribution, and environment health risk of soil HMs in the industrial plants of Xuzhou. Consequently, it is of great significance to investigate the pollution level, identify the fundamental sources, and assess the potential health risks of soil HMs in industrial plants. This study can be used as a reference by other similar cities around the world.

In this study, based on on-site soil samplings and lab experiments in four industrial plants in Xuzhou, the objectives were (1) to investigate the pollution level of HMs using the single factor contaminant index and the Nemerow pollution index, (2) to present the spatial distribution characteristics of HMs, and (3) to quantify the environmental health risk utilizing a USEPA recommended model. The presented results will be helpful for both environmental administration officers and factory managers to prevent soil HM pollution by industrial plants in similar medium-sized cities.

## 2. Materials and Methods

### 2.1. Study Area

**Figure 1:**
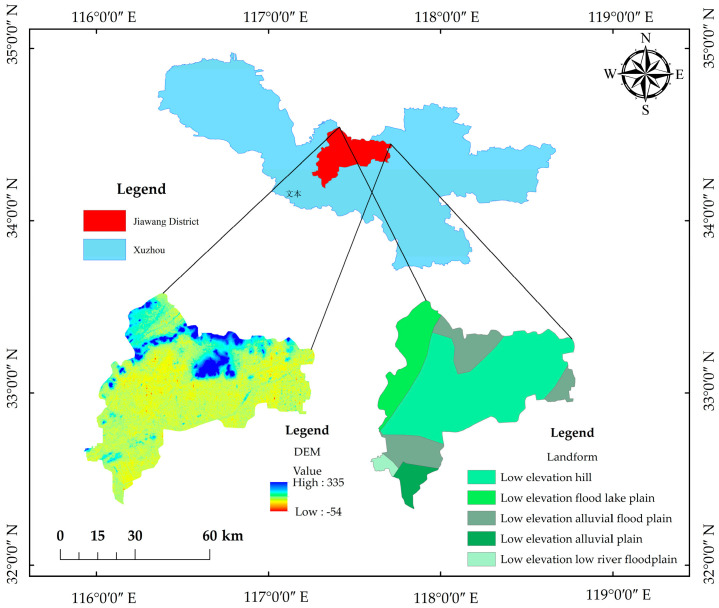
Digital elevation and geomorphology information map.

The study area was located in Xuzhou (33°43′01″–34°58′34″ N, 116°21′29″–118°40′01″ E), Jiangsu Province, with alternating plains and mountains. The plain area accounts for 90%, with the elevation being 20–50 m, while the mountain area accounts for 10%, with the elevation generally being between 100 and 300 m. Xuzhou has a warm temperate monsoon climate, with an annual sunshine duration of 2284–2495 h, an annual average temperature of 14 °C, an annual frost-free period of 200–220 days, and annual average precipitation of 800–930 mm. The climate is characterized by four distinct seasons, abundant sunshine, moderate rainfall, and similar periods of rain and heat. The basic parameters of the soil in the study area are presented in Table 1. The digital elevation and geomorphological information of the study area are shown in Figure 1.

One pharmaceutical plant (A), one iron-steel plant (B), and two chemical plants (C and D) were selected in the study area in Jiawang County, Xuzhou, representing different types of industry. The main soil type in the study area is cinnamon soil.

The annual production capacity of the pharmaceutical plant (A) is 199.48 tons. The main raw and auxiliary materials include ether, ethanol, toluene, dichloromethane, methanol, chloroform, hydrochloric acid, sodium hydroxide, and so on. The iron-steel plant (B) has an annual production capacity of 600,000 tons of continuous casting slab and 900,000 tons of hot-rolled wire rod. The chemical plant (C) has an annual production capacity of 80,000 tons of rutile titanium dioxide, 250,000 tons of by-product ferrous sulfate, and 300,000 tons of sulfur to produce sulfuric acid. The main raw and auxiliary materials include ilmenite, iron powder, caustic soda, hydrochloric acid, limestone powder, sulfur, and so on. The chemical plant (D) is one of the largest producers of vat dyes in China. It has 8 categories and 31 products. The main products are vat dyes and the designed capacity is 9550 tons per year. The main raw and auxiliary materials include anthraquinone, insurance powder, soda ash, sodium acetate, 3,9-dibromobenzoanthrone, benzanthrone, sulfuric acid, hydrochloric acid, chlorobenzene, triethylene glycol, toluene, 1,2-dichloroethane, and lignin.

### 2.2. The Sampling Layout Scheme

**Figure 2:**
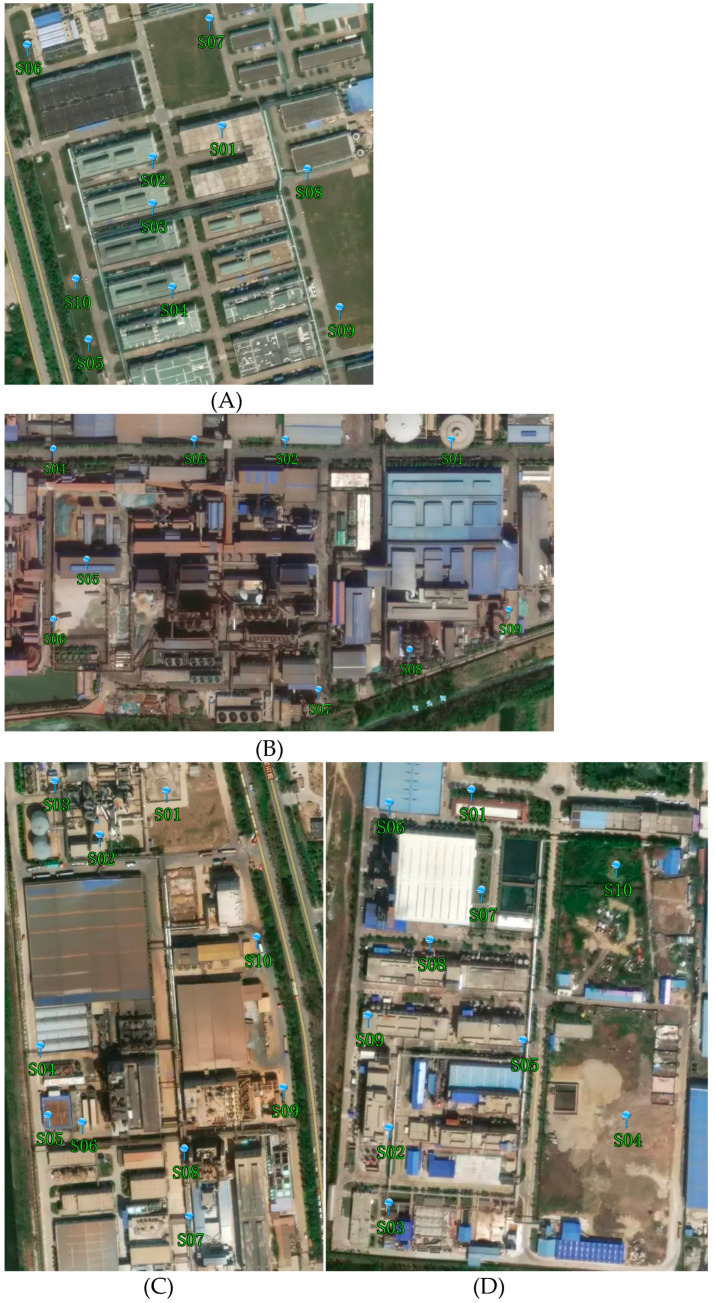
The sampling points at the (**A**–**D**) industrial plants.

Based on previous data collection and an on-site survey, and according to the Technical Guidelines for Environmental Site Investigation, Technical Guidelines for Environmental Site Monitoring [10], and Technical Guidelines of Soil and Groundwater Self-Monitoring for Enterprises In Production [45], the sample points were arranged in key areas, such as the production area, sewage treatment area, hazardous chemical warehouse, calcination area, and warehouse. There were 10 soil sampling points in plants A, C, and D, and 9 soil sampling points in plant B. The sampling points at the four plants are shown in Figure 2.

Soil samples were collected at the target depth using a Geoprobe drilling rig (6610DT) into a PE sampling tube with a length of approximately 20–30 cm. The two ends of the sampling tube were sealed and labeled. Soil samples from the A, B, and C plants were collected on 11–12 July 2018, while those of the D plant were collected on 30 March 2018, and on 18–19 July 2018. Samples at depths of 1 m, 3 m, and 5 m were collected at each soil sampling point, and the depths of S01–S03 and S05 in plant D were different (S01: 1 m, 3 m, 6 m; S02: 1 m, 2.5 m, 4.5 m; S03: 0.5 m, 2.5 m, 4.5 m; S05: 1 m, 2.5 m, 4.5 m). In total, 30, 27, 30, and 30 on-site soil samples were collected from the A, B, C, and D plants, and a total of 117 soil samples were obtained.

### 2.3. Laboratory Testing and Analysis

After sample collection, samples were immediately stored in a 0–4 °C refrigerator and sent to the laboratory within 48 h. Laboratory testing and analysis was carried out at the laboratory of the Jiangsu Fangzheng Environmental Protection Group Co., Ltd. To reduce the impacts of the process of sample preservation and transportation, 2–3 cm soil at the bottom of each sampling tube was removed and 10–60 g soil at the bottom of each sample was taken for testing and analysis. According to “Soil and Sediment-Determination of Aqua Regia Extracts of 12 Metal Elements-Inductively Coupled Plasma Mass Spectrometry” [46], the inductively coupled plasma mass spectrometry method with aqua regia treatment was used for the determination of Cu, Pb, Zn, Ni, Cr, Cd, and As. According to “Soil Quality-Analysis of Total Mercury, Arsenic and Lead Contents-Atomic Fluorescence Spectrometry” [47], the atomic fluorescence spectrometry method was used for the determination of Hg. All metal data mentioned in this study refer to total contents. At least two laboratory blank samples were prepared for each batch of samples, to ensure that the determination was accurate. The correlation coefficient of the standard curve established for each analysis was greater than 0.999. Each batch of samples was tested with parallel double samples and spiked recovery samples at a proportion of at least 10%. Under constant conditions, the relative deviation of the two independent determination results obtained was no more than 12%. Information regarding accuracy is presented in Table 2.

### 2.4. Pollution Level Evaluation Method

Both the single-factor contaminant index *P_i_* and the Nemerow pollution index *P_N_* were used to evaluate the HM pollution level in soil [48,49]. *P_i_* and *P_N_* were calculated using Equations (1) and (2), respectively.

(1) $$P_{i}=\frac{C_{i}}{S_{i}}$$

where *P_i_* (unitless) is the single-factor contaminant index, *C_i_* (mg·kg^−1^) is the detected concentration of each HM *i*, and *S_i_* (mg·kg^−1^) is the evaluation criterion of each HM *i*. The evaluation criterion of this study was the risk screening value (RSV) for soil contamination of agricultural land [11]. The *P_i_* value was classified as safety (*P_i_* ≤ 1), slight pollution (1 < *P_i_* ≤ 2), low pollution (2 < *P_i_* ≤ 3), moderate pollution (3 < *P_i_* ≤ 5), and high pollution (*P_i_* > 5).

(2) $$P_{N}=\sqrt{\frac{{{(P}_{i\max})}^{2}+{\left(P_{iave}\right)}^{2}}{2}}$$

(3) $$P_{iave}=\frac{1}{n}\sum_{i=1}^{n}P_{i}$$

where *P_N_* (unitless) is the Nemerow pollution index, *P_imax_* is the maximum value of *P_i_*, and *P_iave_* is the arithmetic mean value of *P_i_*. The *P_N_* value was classified as safety (*P_N_* ≤ 0.7), warning (0.7 < *P_N_* ≤ 1), slight pollution (1 < *P_N_* ≤ 2), moderate pollution (2 < *P_N_* ≤ 3), and heavy pollution (*P_N_* > 3) [50].

### 2.5. Exposure Assessment of HMs

Exposure assessment of HMs can be performed through direct oral ingestion of the soil (*OISER_j_*), dermal absorption via soils adhered to exposed skin (*DCSER_j_*), and the inhalation intake of resuspended soil particulates (*PISER_j_*) [1]. Three types of exposure dose were calculated using Equations (4)–(6), respectively [51].

(4) $${OISER}_{j}=\frac{{OSIR}_{a}\times {ED}_{a}\times {EF}_{a}\times {ABS}_{o}}{{BW}_{a}\times {AT}_{j}}\times 10^{-6}$$

(5) $${DCSER}_{j}=\frac{{SAE}_{a}\times {SSAR}_{a}\times {EF}_{a}\times {ED}_{a}\times E_{v}\times {ABS}_{d}}{{BW}_{a}\times {AT}_{j}}\times 10^{-6}$$

(6) $${PISER}_{j}=\frac{{PM}_{10}\times {DAIR}_{a}\times {ED}_{a}\times PIAF\times (f_{spo}\times {EFO}_{a}+f_{spi}\times {EFI}_{a})}{{BW}_{a}\times {AT}_{j}}\times 10^{-6}$$

where the subscript *j* represents a noncarcinogenic (nc) or carcinogenic (ca) effect, the subscript *a* represents adults, *OSIR_a_* (mg·d^−1^) is the ingestion rate, *ED_a_* (year) is the exposure duration, *EF_a_* (d·year^−1^) is the exposure frequency, *ABS_o_* (unitless) is the oral absorption factor, *BW_a_* (kg) is the body weight, *AT_j_* (d) is the average exposure time for adults in their whole lifetime, *SAE_a_* (cm^2^) is the exposed skin surface area, *SSAR_a_* (mg·cm^−2^) is the adherence rate of soil on skin, *E_v_* (unitless) is the daily exposure frequency of the dermal contact event, *ABS_d_* (unitless) is the dermal absorption factor, *PM_10_* (mg·m^−3^) is the content of inhalable particulates in ambient air, *DAIR_a_* (m^3^·days^−1^) is the daily air inhalation rate of adults, *PIAF* (unitless) is the retention fraction of inhaled particulates in the body, *f_spo_* (unitless) is the fraction of soil-borne particulates in outdoor air, *f_spi_* (unitless) is the fraction of soil-borne particulates in indoor air, *EFO_a_* (d·a^−1^) is the outdoor exposure frequency, and *EFI_a_* (d·a^−1^) is the indoor exposure frequency.

### 2.6. Health Risk Assessment

The noncarcinogenic effect risk was assessed using the hazard quotient (*HQ*, including *HQ_ois_*, *HQ_dcs_*, and *HQ_pis_*), describing three noncarcinogenic risk indices. They were calculated using Equations (7)–(11) [52].

(7) $${HQ}_{ois}=\frac{{OISER}_{nc}\times C_{sur}}{{RfD}_{o}\times SAF}$$

(8) $${HQ}_{dcs}=\frac{{DCSER}_{nc}\times C_{sur}}{{RfD}_{d}\times SAF}$$

(9) $${RfD}_{d}={RfD}_{o}\times {ABS}_{gi}$$

(10) $${HQ}_{pis}=\frac{{PISER}_{nc}\times C_{sur}}{{RfD}_{i}\times SAF}$$

(11) $${RfD}_{i}=\frac{RfC\times {DAIR}_{a}}{{BW}_{a}}$$

where *HQ_ois_*, *HQ_dcs_*, and *HQ_pis_* are hazard quotients via the three pathways, respectively. *C_sur_* (mg·kg^−1^) is the mean concentration of each HM in the soil samples; *SAF* (unitless) is the soil allocation factor; *RfD_o_*, *RfD_d_*, and *RfD_i_* (mg·kg^−1^·d^−1^) are the corresponding reference doses via different pathways; *ABS_gi_* (unitless) is the digestive tract absorption efficiency factor, and *RfC* (mg·m^−3^) is the respiratory inhalation reference concentration.

The hazard index (*HI*) is equal to the sum of *HQ* and was used to estimate the total noncarcinogenic effects created by all HMs and exposure pathways. If the *HI* value is more than 1, there is a chance that an adverse noncarcinogenic health effect will occur [53].

*CR_ois_*, *CR_dcs_*, and *CR_pis_* describing the carcinogenic risk indices via three pathways were calculated by Equations (12)–(16).

(12) $${CR}_{ois}={OISER}_{ca}\times C_{sur}\times {SF}_{o}$$

(13) $${CR}_{dcs}={DCSER}_{ca}\times C_{sur}\times {SF}_{d}$$

(14) $${SF}_{d}=\frac{{SF}_{o}}{{ABS}_{gi}}$$

(15) $${CR}_{pis}={PISER}_{ca}\times C_{sur}\times {SF}_{i}$$

(16) $${SF}_{i}=\frac{IUR\times {BW}_{a}}{{DAIR}_{a}}$$

where *SF_o_*, *SF_d_*, and *SF_i_* (mg·kg^−1^·d^−1^) are the carcinogenic slope factor via the three pathways, and *IUR* (m^3^·mg^−1^) is the breathing inhalation carcinogen unit. For a single metal, this is negligible when *CR* is less than 10^−6^, while it is unacceptable when CR is more than 10^−4^ [54]. For all the carcinogenic HMs or exposure pathways, the acceptable level of accumulated carcinogenic risk is less than 10^−5^ [55]. Since the health effect mechanism of Pb is different from the others, the health risk for Pb was not considered in this study. Table 3 shows the parameters of the health risk assessment model in this study.

### 2.7. Statistical Analysis

Descriptive statistical indices including the mean, sample variance (SV), sample standard deviation (SSD), coefficient of variation (CV), kurtosis, and skewness were used. CV ≤ 20%, 20% < CV ≤ 50%, 50% < CV ≤ 100%, and CV > 100% indicated low, moderate, high, and exceptionally high variability, respectively [50]. Pearson coefficient correlation analysis was used to investigate the potential pollution sources (industrial activities) of HMs. If there is a significant positive correlation between HMs in soils, they have a high possibility of possessing similar sources.

## 3. Results and Discussion

### 3.1. The Descriptive Statistics of HMs in Soil Profiles

Table 4 shows the descriptive statistical characteristics of HMs in the soil profiles. The HM content at the three depths was highly variable and most CVs were more than 20% and less than 50%, showing moderate variability, except for Hg at a 1 m depth and Pb, Ni, and As at 5 m depth. This can probably be attributed to the different HM background contents and industrial activities.

The mean values of HM contents at different depths were as follows: At 1 m depth, Cu, Pb, Zn, Ni, Cd, and As were 1.30, 1.25, 1.13, 1.07, 3.50, and 1.18 times the BV. At 3 m depth, Cu, Pb, Zn, Ni, Cd, and As were 1.35, 1.41, 1.20, 1.32, 3.70, and 1.38 times the BV. At 5 m depth, Cu, Pb, Zn, Ni, Cd, and As were 1.13, 1.66, 1.09, 1.34, 4.20, and 1.11 times the BV. With the exception of Cr and Hg, the mean values of the above six HMs exceeded the BV. The enrichment of Cd for the three depths exceeded the RSV.

From the average value of the soil profile (AVSP), the content of HMs was highly variable as well. With the exception of Zn, Cr, Hg, and Cd, the AVSPs of the other four HMs were more than the BG and less than the RSV. The AVSPs of Cu, Pb, Ni, Cd, and As were 1.18, 1.42, 1.19, 3.80, and 1.13 times the BG. The maximum values of Pb, Ni, and Cd all exceeded the RSV, and the maximum value of Cd was 6.93 times the RSV. The AVSP of Cd was more than the RSV in the soil profile. This indicates that Cd exceeded its RSV value and may pose a risk to human health.

The order of the CVs of HMs was Hg (103.26%) > Pb (59.65%) > Cd (59.37%) > Zn (56.40%) > Cr (54.02%) > As (50.51%) > Ni (48.02%) > Cu (42.23%). While Hg showed exceptionally high variability, the other seven HMs showed moderate to high variability. This might be due to the influence of different degree of chemical processes on the soil HM pollution in the studied area. It can be seen that Hg, Pb, Cd, and Ni had a positive skewness, with a large value in Table 4. Similar studies showed that HMs have positive skewness if they are disturbed by human activities.

### 3.2. The Descriptive Statistics of HMs in the Different Industrial Plants

In Table 5, the mean values of Cu, Pb, Zn, Ni, Cr, Cd, and As for all depths are higher than the BV in A plant; As and Cd for all depths were higher than the BV in B plant; Cu, Pb, Zn, Ni, Cd, and As for all depths were higher than the BV in C plant; and Cd and Hg for all depths were higher than the BV in D plant. This indicates that many HMs were enriched to some extent in the four industrial plants. Cd in A, B, and C and at 4.5–6 m depth in D exceeded the RSV, indicating that Cd pollution occurred in all four industrial plants. Generally, the enrichment of soil HMs at different depths was mainly concentrated in the A and C industrial plants. This indicates that A and C probably cause greater HM pollution than B and D. The Hg at 5 m depth in the A plant, and Zn at 5 m depth, Ni at 1 m depth, and Cr at 1 m depth in the C plant, showed low variability. In comparison, Cd at 2.5–3 m depth, and Zn and As at 4.5–6 m depth, in the D plant, showed high variability. The rest showed moderate variability. Generally, most HMs showed a moderate variability in all the industrial plants in this study.

### 3.3. Vertical Distribution Characteristics of Soil HMs in the Four Industrial Plants

**Figure 3:**
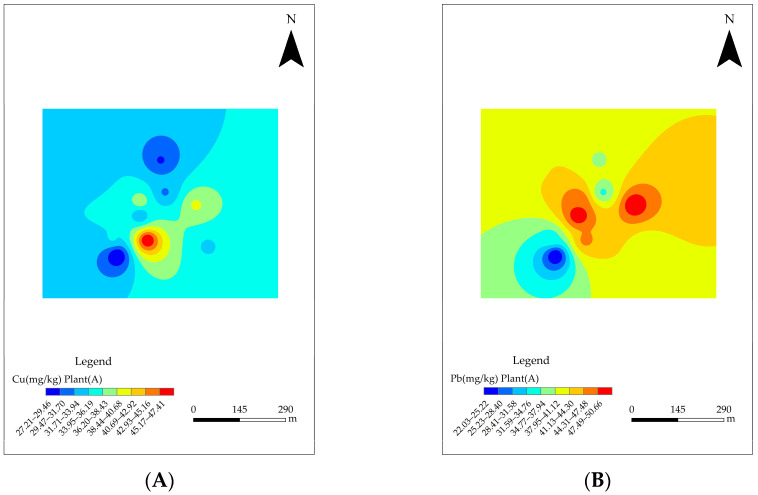

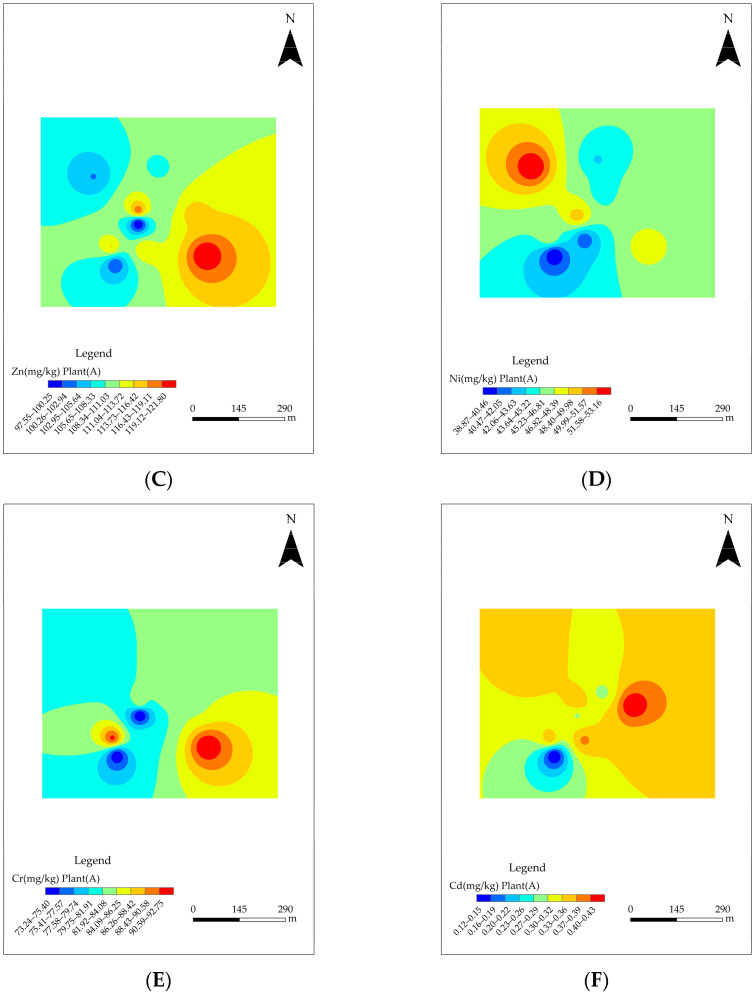

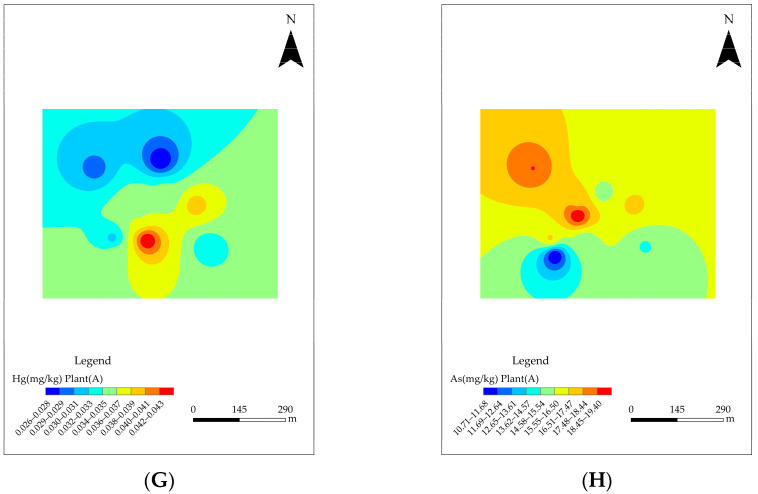
Spatial distribution characteristics of HMs in industrial plant A. (**A**–**H**) represents Cu, Pb, Zn, Ni, Cr, Cd, Hg, and As, respectively.

Figure 3 and Figure S1 show the vertical distribution of the content of the eight HMs in the different industrial plants. Comparisons were made between different layers for the same HM. It should be noted that the 5 m depth layer at the S08 site in industrial plant C was polluted by Pb and Ni through industrial activities. The content of Pb and Ni at a 5 m depth was higher than that of the upper two layers, indicating that Pb and Ni had migrated from the surface to the 5 m depth layer. HM pollution in deep soil may endanger the quality of the groundwater environment. The content of Hg at 1 m depth at S09 in B and at S03 in D was higher than the BV of Hg (0.04 mg·kg^−1^). Hg was introduced into certain sites through industrial activities in the B and D industrial plants.

The content of Cd at most sites of the four industrial plants exceeded not only the BV (0.1 mg·kg^−1^) but also the RSV (0.3 mg·kg^−1^), including S01, S02, S04, and S06–S08 in A, S01–S09 in B, S01–S04 and S06–S10 in C, and S07–S09 in D, which can be seen in Table 5. The content of Cd at the S02 site in the D region even exceeded 2.0 mg·kg^−1^. Cd-induced pollution occurred in all industrial plants.

### 3.4. Spatial Distribution of HMs in Four Industrial Plants

It can be seen that Cu and Hg increased from north to south, and the content was the highest at the S04 site located between the No. 5 and No. 6 synthesis workshops, as shown in Figure 3. This high content of Cu and Hg might be related to industrial activities. Generally, with the exception of Ni and As, the No. 5 and No. 6 synthesis workshops and the dangerous goods warehouse had a strong effect on the content of the HMs.

With the exception of Hg and Pb, the content of the other six HMs in the west was relatively higher than that in the east, as shown in Figure S2A. All HMs had a high content around the S07 and S08 sites in the southeast, whose sites were located near the calcination section in the C plant, as shown in Figure S2B. High contents of Cu, Zn, and Ni were concentrated in the S01 site, surrounding the chemical laboratory building, while the high content of Hg was concentrated in S03 and S04, surrounding the dangerous goods warehouse and sewage treatment station, as seen in Figure S2C.

The sequence of the average content of Cu and Ni was C > A > B > D. The sequence of the average content of Pb, Cr, and Zn was A > C > B > D. The sequence of the average content of Hg was D > A > B > C, while that of As was A > B > C > D. As for Cd, the proportion of sites exceeding the RSV was as high as 79.5% overall.

Three possible reasons may explain the different spatial distribution characteristics of the different HMs. The first reason is that the raw materials and products in different industries were different, causing the types and content of HMs to differ. The second reason might be that the contamination times of soils in the different industrial plants varied. Different HM elements have different migration and adsorption characteristics.

### 3.5. Correlation Analysis of HMs in the Soil

Table 6 shows the Pearson coefficient correlation analysis of HMs in the soils. The correlation between HMs could effectively indicate the sources of HMs. There were significant positive correlations between Cu and Pb, Ni, Cr, and As, with correlation coefficients of 0.364, 0.523, 0.577, and 0.418, respectively. The correlation coefficients between Pb and Ni, Cr, Cd, and As were 0.685, 0.422, 0.416, and 0.323, respectively. The correlation coefficients of Ni with Cr and As were 0.656 and 0.556, respectively. The correlation coefficient between Cr and As was 0.546. From the above, the correlation coefficients of Cu-Ni (0.523), Cu-Cr (0.577), Pb-Ni (0.685), Ni-Cr (0.656), and Ni-As (0.556) were relatively high, indicating that they might have the same sources. Cd was only positively correlated with Pb, and the correlation coefficient was only 0.416, indicating that the Cd might have a single pollution source.

### 3.6. Single Pollution Index of Surface Soil in the Different Industrial Plants

The average single pollution indices of Cd in the A, B, and C industrial plants were more than 1 and less than 2, which indicated a low pollution level, while the other seven HMs belonged to the safe category, as seen in Table S1. Most CVs of the single pollution index of HMs in the A and B industrial plants were more than 20% and less than 50%, which showed moderate variability. Only the CVs of the single pollution indices of Zn, Ni, and Cr in industrial plant C were less than 20%, showing low variability, and those of the other five HMs showed moderate variability. For industrial plant D, the average single pollution indices of all HMs were less than 1 and at a safe level, indicating that the soil in industrial plant D was generally good. The average values of the single pollution indices of Cu, Pb, Zn, Ni, Cr, Cd, Hg, and As in the four plants were 0.31, 0.21, 0.30, 0.34, 0.26, 1.11, 0.02, and 0.44, respectively (see Table S1). There were 28 slightly contaminated sites of Cd, accounting for 71.79%.

### 3.7. The Nemerow Pollution Index of Surface Soil in the Different Industrial Plants

In Table 7, the mean values of the Nemerow pollution index in the A, B, C, and D industrial plants were 0.86, 0.90, 0.94, and 0.70, respectively, which were all more than 0.7 and less than 1.0, corresponding to the warning level. All maximum values in the four industrial plants were more than 1.0, indicating that some sites in each plant had a slight pollution level. The CVs of the Nemerow pollution index in the A, B, C, and D industrial plants were 38.01%, 16.32%, 21.09%, and 48.17%, respectively, with low to moderate variability.

The mean value of the Nemerow pollution index of all industrial plants was 0.85, showing a warning level, with the maximum Nemerow pollution index and CV being 1.40 and 32.22%, respectively. As seen in Table 8, there were 10, 19, and 10 sites with a safe level, warning level, and slight pollution level, accounting for 25.64%, 48.72%, and 25.64% of the 39 sites in the four industrial plants.

### 3.8. Noncarcinogenic Effect in Different Industrial Plants

Table S2 shows the *HQs* and *HIs* of soil HMs under different exposure pathways at the different industrial plants. The *HQs* of Cr and As were higher than those of the other HMs at all industrial plants. As seen in Table 9, the health risk of As via the direct oral ingestion and inhalation intake of resuspended soil particulates reached 51.75% and 47.95%, respectively, and that of Cr through inhalation intake of resuspended soil particulates reached 53.08%. However, the average *HQ_ois_*, *HQ_dcs_*, and *HQ_pis_* were all less than 1, indicating that the Cr and As in all industrial plants did not pose potential noncarcinogenic health risks to humans. In addition, the noncarcinogenic risks of Cu, Zn, and Hg were mainly through direct oral ingestion, while those of Cd and Ni were mainly through the inhalation intake of resuspended soil particulates.

The average HI_s_ of soil HMs for the four industrial plants were all less than 1. The order of the HI values of soil HMs was As > Cr > Ni > Cd > Cu > Zn > Hg.

### 3.9. Carcinogenic Effect in Different Industrial Plants

Table 10 shows the health risk assessment of the carcinogenic effects of soil HMs under different exposure pathways at the different industrial plants. The CRs of Cr in plants A and C were unacceptable (Table 10). The average CRs of Cr, As, Cd, and Ni in the four industrial plants were 9.69 × 10^−5^, 9.48 × 10^−6^, 2.59 × 10^−6^, and 1.37 × 10^−5^, respectively, with an order of Cr > Ni > As > Cd. The carcinogenic effect of Cr through the inhalation intake of resuspended soil particulates reached 86.37%, and the carcinogenic effect of Cd, Ni, and As via direct oral ingestion reached 81.06%, 86.44%, and 87.83%, respectively (Table 7).

## 4. Conclusions

This study showed that the HMs at all depths were highly variable, and most CVs showed moderate variability. Cd at three depths even exceeded the RSV. Hg, Pb, Cd, and Ni might have been disturbed by industrial activities. HMs were enriched at all four industrial plants. Cd pollution occurred at all industrial plants. Different HMs had different vertical distribution characteristics. The raw materials and products not only made the soil HM spatial distribution characteristics different but caused the metal types and contents to be different. The correlation analysis of soil HMs suggested that Cu-Ni, Cu-Cr, Pb-Ni, Ni-Cr, and Ni-As might have the same sources. Cd might have a single pollution source. More attention should be paid to the raw materials during the production process to minimize HM pollution. The average single pollution indices of Cd at the A, B, and C industrial plants implied a slight pollution level, which indicated that Cd posed a possible risk to human health. The other seven HMs belonged to the safe category. The *HQ_s_* of Cr and As were higher than the those for other HMs and were all less than 1, indicating that Cr and As did not pose potential noncarcinogenic health risks to humans at all the industrial plants for the area investigated in this study.

## Figures and Tables

**Table 1:** The basic parameters of the soil in the study area.

HM	Cu	Pb	Zn	Ni	Cr	Cd	Hg	As
BV (mg/kg)	24.30	21.30	74.10	30.70	64.80	0.10	0.04	11.60
pH	5.50							
Moisture content (%)	12.35							

BV: Background value [41,42]. pH: Soil—determination of pH—potentiometry [43]. Moisture content was determined on-site using a three-parameter soil speed tester. Instrument model: Delta-T ML3-KIT [44].

**Table 2:** The parameters of laboratory testing and analysis [46].

HM		Cu	Pb	Zn	Ni	Cr	Cd	Hg	As
Hot plate	Detection limit (mg/kg)	0.5	2	7	2	2	0.07	_	0.6
	Determination limit (mg/kg)	2.0	8	28	8	8	0.28	_	2.4
Microwave	Detection limit (mg/kg)	0.6	2	1	1	2	0.09	_	0.4
	Determination limit (mg/kg)	2.4	8	4	4	8	0.36	_	1.6
Atomic fluorescence	Detection limit (mg/kg)	_	_	_	_	_	_	0.002	_

**Table 3:** Parameters of the human health risk assessment model in this study.

Parameter/Unit	Value	Parameter/Unit	Value	Parameter/Unit	Value
*OSIR_a_* (mg·d^−1^)	100	*H_a_* (cm)	156.3	*f_spi_*	0.8
*ED_a_* (a)	25	*SER_a_*	0.18	*f_spo_*	0.5
*EF_a_* (d·a^−1^)	250	*ABS_d_*	0.001	*EFI_a_* (d·a^−1^)	187.5
*BW_a_* (kg)	56.8	*Ev* (times·d^−1^)	1	*EFO_a_* (d·a^−1^)	62.5
*ABS_o_*	1	*PM10* (mg·m^−3^)	0.15	*SAF*	0.2
*ATca* (d)	26280	*DAIR_a_* (m^3^·d^−1^)	14.5	*SSAR_a_* (mg·cm^−2^)	0.2
*ATnc* (d)	9125	*PIAF*	0.75		
	***SF_o_* mg·kg^−1^·d^−1^**	***ABS_gi_***	***RfD_o_* mg·kg^−1^·d^−1^**	***RfC* mg·m^−3^**	***IUR* m^3^·mg^−1^**
Cu	/	1.00	4.00 × 10^−2^	/	/
Zn	/	1.00	3.00 × 10^−1^	/	/
Ni	8.40 × 10^−1^	4.00 × 10^−2^	2.00 × 10^−2^	9.00 × 10^−5^	2.60 × 10^−1^
Cr	5.00 × 10^−1^	2.50 × 10^−2^	3.00 × 10^−3^	1.00 × 10^−4^	8.40
Cd	1.50	2.50 × 10^−1^	1.00 × 10^−3^	1.00 × 10^−5^	1.80
Hg	/	7 × 10^−2^	3.00 × 10^−4^	3.00 × 10^−4^	/
As	1.50	1	3.00 × 10^−4^	1.50 × 10^−5^	4.30

**Table 4:** Descriptive statistical characteristics of HMs in the soil profiles.

Column	HM	Mean mg·kg^−1^	Min mg·kg^−1^	Max mg·kg^−1^	SD	CV %	Kurtosis	Skewness	BV mg·kg^−1^	RSV mg·kg^−1^	RIV mg·kg^−1^
1st/1.0 m	Cu	31.63	12.45	76.99	12.39	39.18	4.19	1.54	24.30	100.00	-
	Pb	26.68	6.20	66.31	13.19	49.43	1.63	1.18	21.30	120.00	700
	Zn	84.00	11.06	128.80	36.78	43.78	−0.09	−1.06	74.10	250.00	-
	Ni	32.98	8.38	51.09	11.44	34.70	−0.51	−0.21	30.70	100.00	-
	Cr	54.01	11.97	92.00	25.61	47.41	−1.32	−0.11	64.80	200.00	1000
	Cd	0.35	-	0.58	0.12	34.52	1.62	−0.71	0.10	0.30	3.0
	Hg	0.04	0.02	0.13	0.02	57.82	6.64	2.41	0.04	2.40	4.0
	As	13.64	4.74	21.30	4.31	31.63	−0.48	−0.02	11.60	30.00	120
2nd/3.0 m	Cu	32.71	20.02	47.40	7.33	22.41	−0.89	0.20	24.30	100.00	-
	Pb	30.03	15.05	71.40	12.13	40.39	3.04	1.42	21.30	120.00	700
	Zn	88.84	9.30	136.38	38.77	43.64	−0.15	−1.04	74.10	250.00	-
	Ni	40.55	3.10	75.19	12.23	30.16	2.87	−0.18	30.70	100.00	-
	Cr	60.57	7.90	100.63	26.01	42.94	−1.18	−0.27	64.80	200.00	1000
	Cd	0.37	-	0.64	0.14	37.26	0.87	−0.28	0.10	0.30	3.0
	Hg	0.04	0.02	0.07	0.01	37.17	0.09	0.91	0.04	2.40	4.0
	As	15.96	1.35	23.44	5.87	36.77	1.27	−1.24	11.60	30.00	120
3rd/5.0 m	Cu	27.43	8.10	53.20	9.85	35.90	1.24	0.55	24.30	100.00	-
	Pb	35.38	8.89	136.67	24.92	70.46	7.89	2.51	21.30	120.00	700
	Zn	80.78	4.30	143.17	37.24	46.10	−0.01	−0.84	74.10	250.00	-
	Ni	41.15	8.42	144.23	23.99	58.31	9.80	2.58	30.70	100.00	-
	Cr	56.80	4.30	102.85	25.36	44.64	−0.70	−0.26	64.80	200.00	1000
	Cd	0.42	0.06	0.79	0.14	33.99	1.04	−0.16	0.10	0.30	3.0
	Hg	0.02	0.01	0.05	0.01	33.72	2.81	0.94	0.04	2.40	4.0
	As	12.89	0.20	29.87	7.62	59.13	−0.37	0.10	11.60	30.00	120
AVSP	Cu	28.72	0.99	76.99	12.13	42.23	1.89	0.50	24.30	100.00	-
	Pb	30.17	4.85	136.67	18.00	59.65	11.06	2.63	21.30	120.00	700
	Zn	74.23	2.06	143.17	41.86	56.40	−1.24	−0.46	74.10	250.00	-
	Ni	36.50	1.24	144.23	17.53	48.02	11.48	2.00	30.70	100.00	-
	Cr	51.76	3.70	102.85	27.96	54.02	−1.25	−0.07	64.80	200.00	1000
	Cd	0.38	-	2.08	0.22	59.37	27.82	3.64	0.10	0.30	3.0
	Hg	0.04	0.01	0.42	0.04	103.26	54.59	6.49	0.04	2.40	4.0
	As	13.05	0.20	29.87	6.59	50.51	−0.45	−0.22	11.60	30.00	120

BV: Background value in Jiangsu [41,42]. RSV: Risk screening value for soil contamination of agricultural land [11]. RIV: Risk intervention value for soil contamination of agricultural land [11]. Soil environmental quality: Risk control standard for soil contamination of agricultural land [11]. AVSP: The average value of the soil profile.

**Table 5:** Descriptive statistical characteristics of HMs in the different industrial plants.

	HM	Depth m	Mean mg·kg^−1^	Min mg·kg^−1^	Max mg·kg^−1^	SD	CV %	Kurtosis	Skewness	BV mg·kg^−1^	RSV mg·kg^−1^	RIV mg·kg^−1^
		1.0	40.12	26.87	50.45	7.91	19.72	−1.14	−0.49	24.30	100.00	-
	Cu	3.0	35.35	25.54	42.49	6.04	17.07	−1.41	−0.50	24.30	100.00	-
		5.0	27.78	9.07	50.31	10.61	38.19	2.33	0.57	24.30	100.00	-
		1.0	42.04	27.12	66.31	12.56	29.88	−0.14	0.69	21.30	120.00	700
	Pb	3.0	38.09	15.05	71.40	15.46	40.59	1.77	1.01	21.30	120.00	700
		5.0	39.99	20.15	78.13	16.89	42.24	2.09	1.20	21.30	120.00	700
		1.0	110.96	128.80	86.59	13.38	12.06	−0.55	−0.65	74.10	250.00	-
	Zn	3.0	112.96	86.30	136.38	16.41	14.53	−0.57	−0.51	74.10	250.00	-
		5.0	106.21	64.21	143.17	21.44	20.19	1.17	−0.21	74.10	250.00	-
		1.0	44.73	27.26	51.09	7.54	16.86	2.61	−1.71	30.70	100.00	-
A	Ni	3.0	52.79	39.79	75.19	9.10	17.24	4.61	1.66	30.70	100.00	-
		5.0	39.94	20.54	62.87	11.84	29.64	0.81	0.32	30.70	100.00	-
		1.0	82.29	62.38	92.00	10.00	12.15	−0.13	−0.77	64.80	200.00	1000
	Cr	3.0	85.86	63.47	100.63	10.27	11.96	1.79	−1.02	64.80	200.00	1000
		5.0	79.48	43.44	102.85	17.09	21.51	1.25	−0.72	64.80	200.00	1000
		1.0	0.33	0.07	0.57	0.15	45.45	−0.38	−0.03	0.10	0.30	3
	Cd	3.0	0.30	0.16	0.38	0.08	26.57	−0.81	−0.74	0.10	0.30	3
		5.0	0.33	0.06	0.56	0.13	39.19	2.34	−0.59	0.10	0.30	3
		1.0	0.04	0.03	0.06	0.01	25.00	4.50	1.54	0.04	2.40	4
	Hg	3.0	0.04	0.02	0.05	0.01	22.74	−0.88	−0.18	0.04	2.40	4
		5.0	0.02	0.02	0.03	0.00	9.96	−1.41	0.68	0.04	2.40	4
		1.0	16.98	10.86	21.30	3.48	20.49	−0.90	−0.46	11.60	30.00	120
	As	3.0	18.27	12.04	22.74	3.34	18.30	−0.40	−0.44	11.60	30.00	120
		5.0	12.50	1.16	29.87	7.51	60.07	3.16	1.23	11.60	30.00	120
		1.0	23.02	18.52	29.20	3.73	16.20	−0.38	0.73	24.30	100.00	-
	Cu	3.0	28.67	23.82	36.35	4.28	14.94	−0.63	0.72	24.30	100.00	-
		5.0	24.05	8.10	32.16	7.01	29.14	3.27	−1.56	24.30	100.00	-
		1.0	17.62	6.20	25.34	6.70	38.01	−0.42	−0.70	74.10	120.00	700
	Pb	3.0	27.80	15.10	50.70	11.09	39.89	1.29	0.92	74.10	120.00	700
		5.0	24.34	8.89	48.24	12.41	50.98	0.39	1.02	74.10	120.00	700
		1.0	25.12	21.09	33.98	4.27	17.01	0.96	1.17	30.70	100.00	-
B	Ni	3.0	34.31	25.55	42.94	5.44	15.87	−0.50	0.13	30.70	100.00	-
		5.0	30.67	20.16	46.76	8.04	26.22	0.91	0.94	30.70	100.00	-
		1.0	31.09	24.14	39.24	5.47	17.59	−1.19	0.23	64.80	200.00	1000
	Cr	3.0	40.32	35.16	48.95	4.13	10.24	1.60	1.12	64.80	200.00	1000
		5.0	39.86	31.55	45.39	4.80	12.04	−1.00	−0.57	64.80	200.00	1000
		1.0	0.37	0.27	0.44	0.06	16.42	−1.09	−0.14	0.10	0.30	3
	Cd	3.0	0.49	0.35	0.64	0.11	22.41	−1.81	0.13	0.10	0.30	3
		5.0	0.55	0.37	0.79	0.11	20.65	2.98	1.01	0.10	0.30	3
		1.0	0.04	0.02	0.12	0.03	67.96	7.35	2.64	0.04	2.40	4
	Hg	3.0	0.03	0.02	0.05	0.01	35.49	0.28	1.13	0.04	2.40	4
		5.0	0.03	0.01	0.03	0.01	34.05	3.46	−1.70	0.04	2.40	4
		1.0	11.99	8.75	20.15	3.44	28.72	4.24	1.91	11.60	30.00	120
	As	3.0	17.91	1.95	23.44	6.62	36.96	4.80	−2.03	11.60	30.00	120
		5.0	15.37	3.46	25.66	7.85	51.08	−0.65	−0.33	11.60	30.00	120
		1.0	36.49	24.19	76.99	14.82	40.61	7.97	2.70	24.30	100.00	-
	Cu	3.0	36.46	29.36	45.77	5.52	15.13	−0.86	0.52	24.30	100.00	-
		5.0	31.08	21.92	53.20	9.46	30.42	2.72	1.70	24.30	100.00	-
		1.0	24.42	16.38	32.33	4.94	20.25	−0.37	0.12	21.30	120.00	700
	Pb	3.0	28.73	15.93	42.43	7.63	26.56	0.02	0.20	21.30	120.00	700
		5.0	48.87	18.35	136.67	36.77	75.25	3.28	1.88	21.30	120.00	700
		1.0	90.24	70.09	110.00	10.50	11.63	1.72	0.11	74.10	250.00	-
	Zn	3.0	99.57	82.62	121.23	15.69	15.76	−1.53	0.54	74.10	250.00	-
		5.0	87.91	76.20	103.80	8.55	9.72	0.07	0.86	74.10	250.00	-
		1.0	36.14	29.44	43.72	3.45	9.55	3.84	0.46	30.70	100.00	-
C	Ni	3.0	41.70	36.53	49.38	4.46	10.69	−0.89	0.71	30.70	100.00	-
		5.0	62.43	36.38	144.23	31.88	51.07	5.35	2.21	30.70	100.00	-
		1.0	64.07	51.48	71.99	5.32	8.30	3.65	−1.36	64.80	200.00	1000
	Cr	3.0	73.72	60.96	88.65	10.73	14.56	−1.55	0.49	64.80	200.00	1000
		5.0	62.43	36.38	144.23	31.88	51.07	5.35	2.21	64.80	200.00	1000
		1.0	0.38	0.25	0.50	0.08	21.89	−0.78	0.42	0.10	0.30	3
	Cd	3.0	0.41	0.26	0.59	0.09	21.64	2.16	0.43	0.10	0.30	3
		5.0	0.46	0.33	0.59	0.09	18.54	−0.93	0.38	0.10	0.30	3
		1.0	0.02	0.02	0.03	0.01	21.32	−1.31	−0.65	0.04	2.40	4
	Hg	3.0	0.03	0.02	0.03	0.01	20.07	0.07	−0.72	0.04	2.40	4
		5.0	0.02	0.01	0.03	0.01	31.58	7.68	2.69	0.04	2.40	4
		1.0	13.56	6.24	19.85	3.62	26.65	1.57	−0.44	11.60	30.00	120
	As	3.0	14.61	1.49	18.68	4.90	33.53	7.09	4.90	11.60	30.00	120
		5.0	15.40	3.52	25.17	5.51	35.77	2.61	−0.62	11.60	30.00	120
		0.5–1.0	24.37	9.20	64.66	15.30	62.78	6.49	2.30	24.30	100.00	-
	Cu	2.5–3.0	16.44	2.53	47.40	13.73	83.53	1.83	1.28	24.30	100.00	-
		4.5–6.0	19.76	0.99	43.61	14.13	71.50	−0.99	0.57	24.30	100.00	-
		0.5–1.0	20.56	9.64	32.69	7.86	38.25	−1.07	−0.09	21.30	120.00	700
	Pb	2.5–3.0	17.23	4.85	24.27	6.52	37.81	−0.05	−0.91	21.30	120.00	700
		4.5–6.0	30.67	13.73	86.12	21.35	69.63	5.78	2.29	21.30	120.00	700
		0.5–1.0	26.86	11.06	51.37	12.33	45.90	0.30	0.60	74.10	250.00	-
	Zn	2.5–3.0	14.06	2.06	25.74	8.90	63.32	−1.59	−0.29	74.10	250.00	-
		4.5–6.0	22.40	2.26	102.26	29.03	129.59	8.27	2.79	74.10	250.00	-
		0.5–1.0	27.99	8.38	58.05	16.80	60.02	−0.58	0.62	30.70	100.00	-
D	Ni	2.5–3.0	20.98	1.24	35.46	13.15	62.67	−1.58	−0.45	30.70	100.00	-
		4.5–6.0	18.69	8.36	30.57	8.77	46.92	−1.62	0.08	30.70	100.00	-
		0.5–1.0	25.98	11.97	68.49	16.19	62.30	6.33	2.29	64.80	200.00	1000
	Cr	2.5–3.0	14.22	3.70	27.87	8.52	59.91	−1.40	0.36	64.80	200.00	1000
		4.5–6.0	12.64	3.89	28.93	7.76	61.35	0.99	0.80	64.80	200.00	1000
		0.5–1.0	0.26	0.00	0.58	0.17	65.51	0.71	−0.04	0.10	0.30	3
	Cd	2.5–3.0	0.13	0.00	0.35	0.15	109.82	−1.41	0.65	0.10	0.30	3
		4.5–6.0	0.57	0.14	2.08	0.56	98.12	6.92	2.51	0.10	0.30	3
		0.5–1.0	0.12	0.04	0.42	0.11	96.96	8.03	2.75	0.04	2.40	4
	Hg	2.5–3.0	0.06	0.04	0.07	0.01	16.74	−0.97	−0.22	0.04	2.40	4
		4.5–6.0	0.04	0.02	0.13	0.03	70.09	6.99	2.50	0.04	2.40	4
		0.5–1.0	10.39	4.74	16.47	3.81	36.62	−0.87	0.04	11.60	30.00	120
	As	2.5–3.0	7.75	0.38	21.18	6.19	79.85	1.41	1.04	11.60	30.00	120
		4.5–6.0	2.99	0.20	14.72	4.38	146.42	7.02	2.53	11.60	30.00	120

**Table 6:** Pearson coefficient correlation analysis of HMs in the soil.

HM	Cu	Pb	Zn	Ni	Cr	Cd	Hg	As
Cu	1.000							
Pb	0.364 **	1.000						
Zn	0.015	0.000	1.000					
Ni	0.523 **	0.685 **	−0.146	1.000				
Cr	0.577 **	0.422 **	0.176	0.656 **	1.000			
Cd	0.034	0.416 **	−0.097	0.077	0.001	1.000		
Hg	0.071	−0.119	0.158	−0.156	−0.285 **	−0.193 *	1.000	
As	0.418 **	0.323 **	0.162	0.556 **	0.546 **	0.087	−0.163	1.000

** Significant correlation at 0.01 level (bilateral). * Significant correlation at 0.05 level (bilateral).

**Table 7:** Nemerow pollution index of soil HMs in the different industrial plants.

	Index	Mean	Min	Max	SD	CV%	Sum
A	P_N_	0.86	0.42	1.40	0.33	38.01	10
B	P_N_	0.90	0.67	1.10	0.15	16.32	9
C	P_N_	0.94	0.63	1.23	0.20	21.09	10
D	P_N_	0.70	0.19	1.39	0.34	48.17	10
All	P_N_	0.85	0.19	1.40	0.27	32.22	39

**Table 8:** Statistics for the Nemerow pollution index of soil HMs.

Grade	*P_N_*	Level	Sum	Percent
1	*P_N_* ≤ 0.7	Safety	10	25.64%
2	0.7 < *P_N_* ≤ 1.0	Warning	19	48.72%
3	1.0 < *P_N_* ≤ 2.0	Slight pollution	10	25.64%
4	2.0 < *P_N_* ≤ 3.0	Moderate pollution	0	0%
5	*P_N_* > 3.0	Heavy pollution	0	0%
-	*P_N_* > 1.0		10	25.64%

**Table 9:** Contribution rate of different exposure pathways of soil HMs in the study area.

	Contribution Rate%	Cu	Zn	Hg	Cd	Ni	Cr	As
	1	-	-	-	81.06	86.44	11.10	87.83
Carcinogenic	2	-	-	-	18.49	12.33	2.53	0.50
	3	-	-	-	0.45	1.24	86.37	11.66
	1	99.43	99.43	88.66	17.06	8.74	38.19	51.75
Noncarcinogenic	2	0.57	0.57	7.23	3.90	1.25	8.72	0.30
	3	-	-	4.11	79.04	90.01	53.08	47.95

1: Direct oral ingestion of the soil. 2: Dermal absorption via the soils adhered to exposed skin. 3: Inhalation intake of resuspended soil particulates.

**Table 10:** Health risk assessment of carcinogenic effects of soil HMs under different exposure pathways at the different industrial plants.

		**A**				**B**			
		**CR_ois_**	**CR_dcs_**	**CR_pis_**	**CR**	**CR_ois_**	**CR_dcs_**	**CR_pis_**	**CR**
Cr	Mean	1.72 × 10^−5^	3.94 × 10^−6^	1.34 × 10^−4^	1.55 × 10^−4^	6.51 × 10^−6^	1.49 × 10^−6^	5.06 × 10^−5^	5.86 × 10^−5^
As	Mean	1.07 × 10^−5^	6.09 × 10^−8^	1.42 × 10^−6^	1.21 × 10^−5^	7.53 × 10^−6^	4.30 × 10^−8^	1.00 × 10^−6^	8.57 × 10^−6^
Cd	Mean	2.05 × 10^−6^	4.68 × 10^−7^	1.14 × 10^−8^	2.53 × 10^−6^	2.32 × 10^−6^	5.29 × 10^−7^	1.29 × 10^−8^	2.86 × 10^−6^
Ni	Mean	1.57 × 10^−5^	2.25 × 10^−6^	2.26 × 10^−7^	1.82 × 10^−5^	8.85 × 10^−6^	1.26 × 10^−6^	1.27 × 10^−7^	1.02 × 10^−5^
		**C**				**D**			
		**CR_ois_**	**CR_dcs_**	**CR_pis_**	**CR**	**CR_ois_**	**CR_dcs_**	**CR_pis_**	**CR**
Cr	Mean	1.34 × 10^−5^	3.06 × 10^−6^	1.04 × 10^−4^	1.21 × 10^−4^	5.44 × 10^−6^	1.24 × 10^−6^	4.24 × 10^−5^	4.90 × 10^−5^
As	Mean	8.52 × 10^−6^	4.86 × 10^−8^	1.13 × 10^−6^	9.71 × 10^−6^	6.52 × 10^−6^	3.73 × 10^−8^	8.67 × 10^−7^	7.43 × 10^−6^
Cd	Mean	2.39 × 10^−6^	5.45 × 10^−7^	1.33 × 10^−8^	2.95 × 10^−6^	1.65 × 10^−6^	3.77 × 10^−7^	9.19 × 10^−9^	2.04 × 10^−6^
Ni	Mean	1.27 × 10^−5^	1.81 × 10^−6^	1.82 × 10^−7^	1.47 × 10^−5^	9.85 × 10^−6^	1.40 × 10^−6^	1.41 × 10^−7^	1.14 × 10^−5^
		**Average of four plants**							
		**CR_ois_**	**CR_dcs_**	**CR_pis_**	**CR**				
Cr		1.08 × 10^−5^	2.46 × 10^−6^	8.37 × 10^−5^	9.69 × 10^−5^				
As		8.33 × 10^−6^	4.76 × 10^−8^	1.11 × 10^−6^	9.48 × 10^−6^				
Cd		2.10 × 10^−6^	4.78 × 10^−7^	1.16 × 10^−8^	2.59 × 10^−6^				
Ni		1.19 × 10^−5^	1.69 × 10^−6^	1.70 × 10^−7^	1.37 × 10^−5^				

## Data Availability

Not applicable.

## Supplementary Materials

The following supporting information can be downloaded at: https://www.mdpi.com/article/10.3390/toxics11030217/s1, Figure S1: Vertical distribution of eight HM contents at different depths in different industrial plants; Figure S2: Spatial distribution characteristics of HMs in four industrial plants; Table S1: The single factor contaminant index of HMs in different industrial plants; Table S2: HQs and HIs of soil HMs under different exposure pathways in different industrial plants.
