# Supplementary material for: Risk Assessment of Heavy Metals in Soils from Four Different Industrial Plants in a Medium-Sized City in North China
Source: Toxics. 2023 Feb 25;11(3):217. doi: 10.3390/toxics11030217 (PMC10059013; doi:10.3390/toxics11030217)
Supplement: Supplementary file 1 [file toxics-11-00217-s001.zip › toxics-2182724-supplementary.pdf]

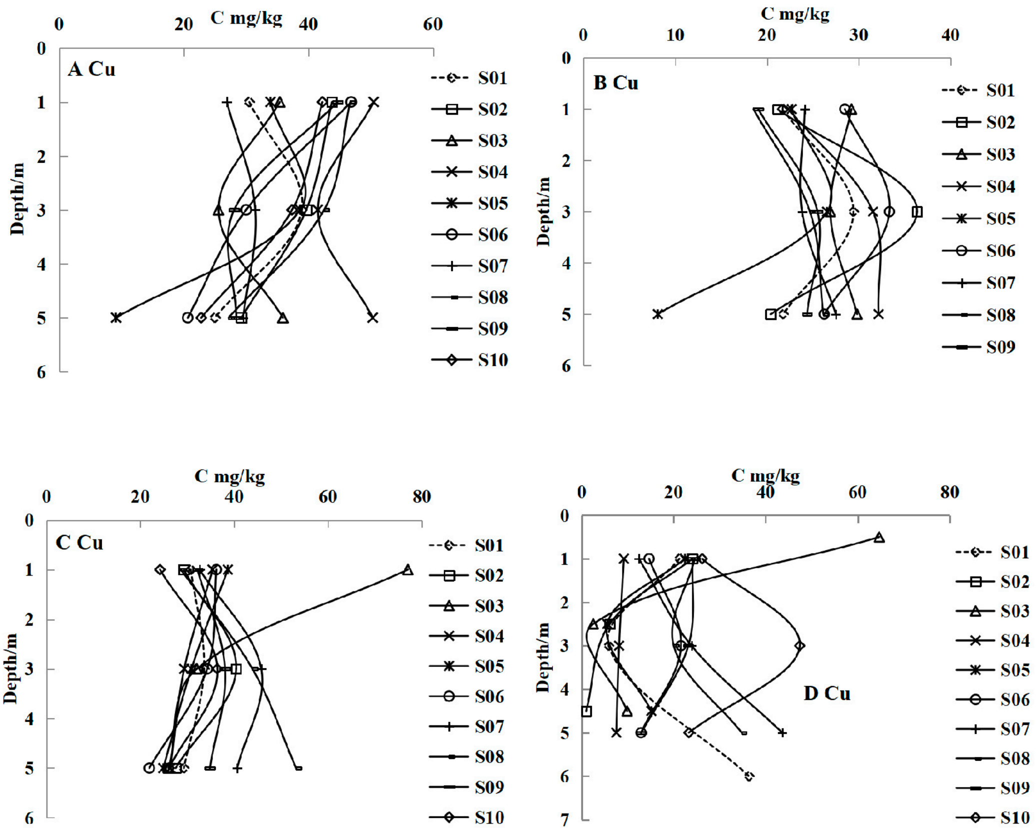
A Cu
B Cu
C Cu
D Cu
C mg/kg
Depth/m
S01
S02
S03
S04
S05
S06
S07
S08
S09
S10

(A)

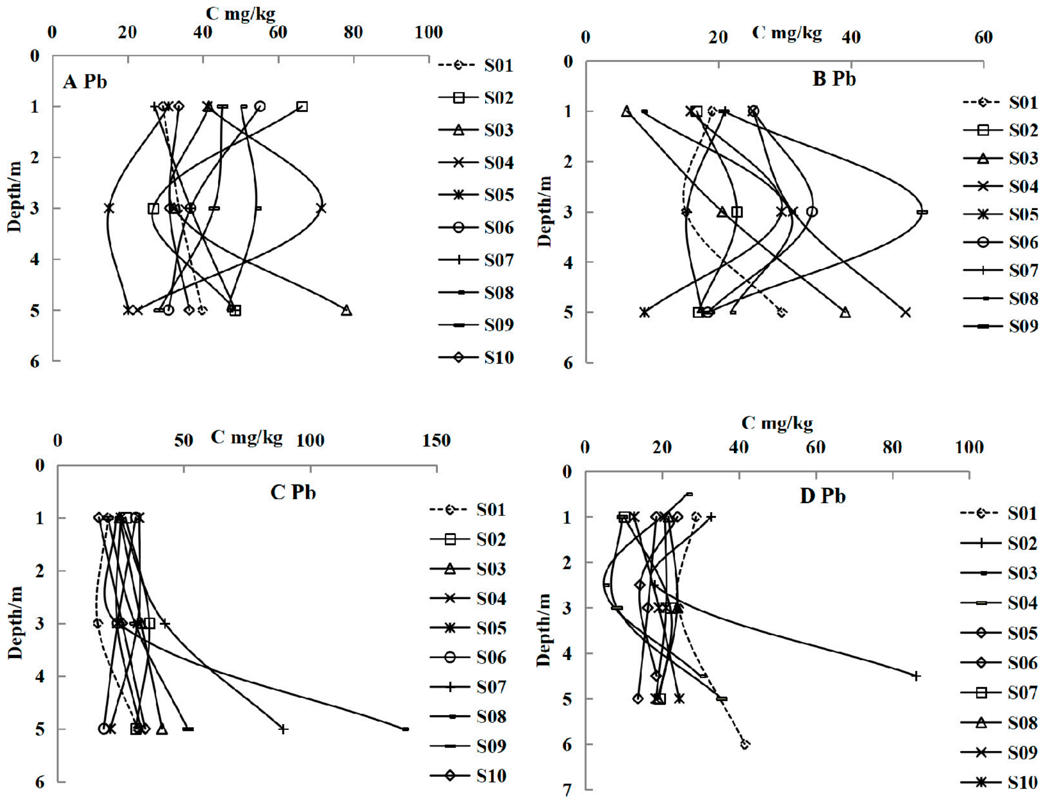
A Pb
B Pb
C Pb
D Pb
C mg/kg
Depth/m
S01
S02
S03
S04
S05
S06
S07
S08
S09
S10

(B)

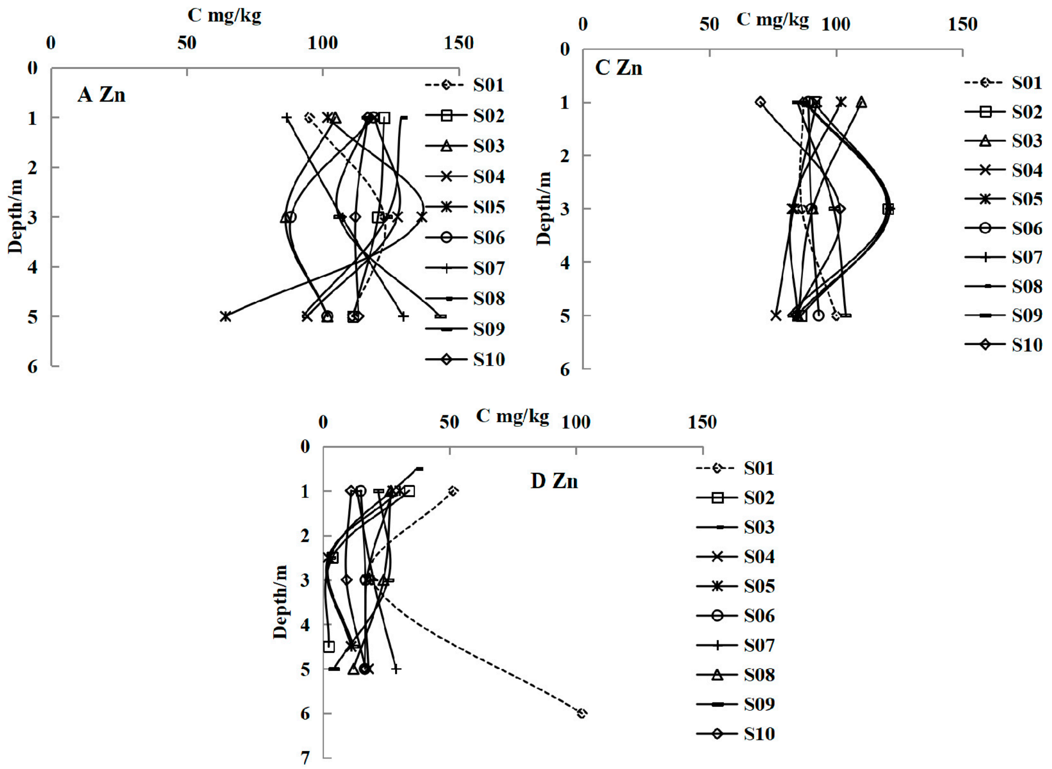

A Zn
C mg/kg
0
50
100
150
Depth/m
0
1
2
3
4
5
6
S01
S02
S03
S04
S05
S06
S07
S08
S09
S10
C Zn
C mg/kg
0
50
100
150
Depth/m
0
1
2
3
4
5
6
S01
S02
S03
S04
S05
S06
S07
S08
S09
S10
D Zn
C mg/kg
0
50
100
150
Depth/m
0
1
2
3
4
5
6
7
S01
S02
S03
S04
S05
S06
S07
S08
S09
S10

(C)

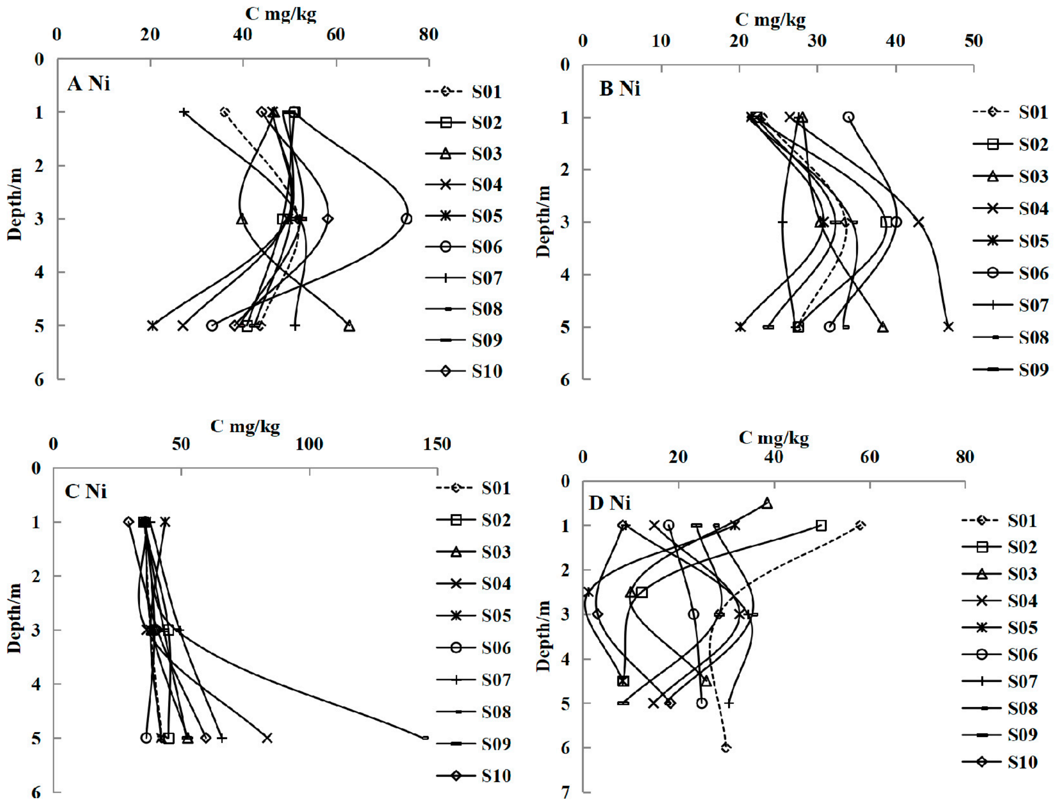
A Ni
C mg/kg
Depth/m
S01
S02
S03
S04
S05
S06
S07
S08
S09
S10
B Ni
C mg/kg
Depth/m
S01
S02
S03
S04
S05
S06
S07
S08
S09
C Ni
C mg/kg
Depth/m
S01
S02
S03
S04
S05
S06
S07
S08
S09
S10
D Ni
C mg/kg
Depth/m
S01
S02
S03
S04
S05
S06
S07
S08
S09
S10

(D)

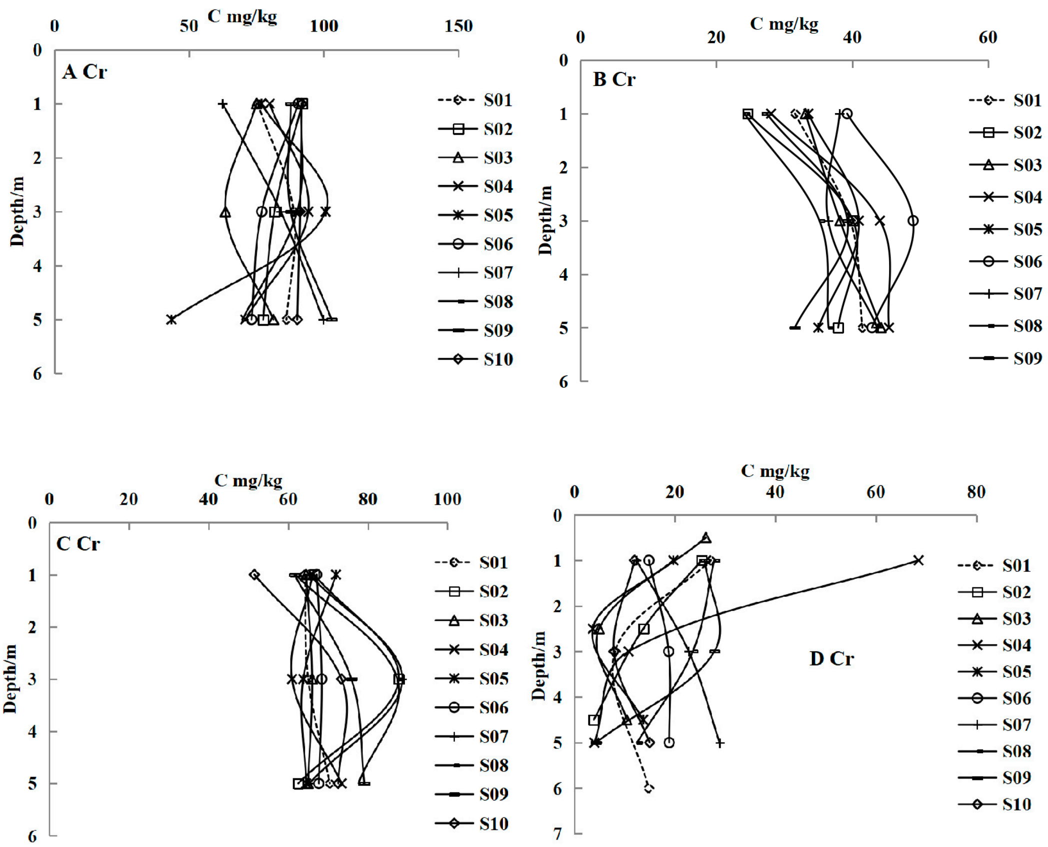
A Cr
C mg/kg
0
50
100
150
Depth/m
0
1
2
3
4
5
6
S01
S02
S03
S04
S05
S06
S07
S08
S09
S10
B Cr
C mg/kg
0
20
40
60
Depth/m
0
1
2
3
4
5
6
S01
S02
S03
S04
S05
S06
S07
S08
S09
C Cr
C mg/kg
0
20
40
60
80
100
Depth/m
0
1
2
3
4
5
6
S01
S02
S03
S04
S05
S06
S07
S08
S09
S10
D Cr
C mg/kg
0
20
40
60
80
Depth/m
0
1
2
3
4
5
6
7
S01
S02
S03
S04
S05
S06
S07
S08
S09
S10

(E)

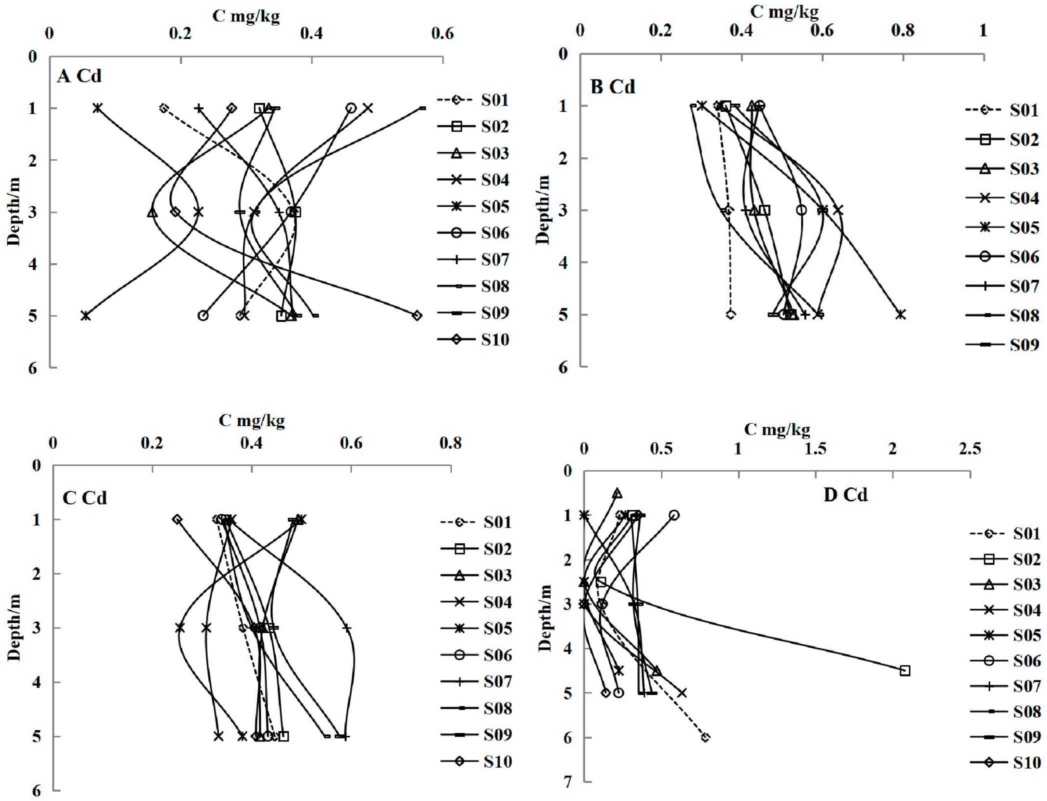

C mg/kg
0
0.2
0.4
0.6
A Cd
Depth/m
S01
S02
S03
S04
S05
S06
S07
S08
S09
S10
C mg/kg
0
0.2
0.4
0.6
0.8
1
B Cd
Depth/m
S01
S02
S03
S04
S05
S06
S07
S08
S09
C mg/kg
0
0.2
0.4
0.6
0.8
C Cd
Depth/m
S01
S02
S03
S04
S05
S06
S07
S08
S09
S10
C mg/kg
0
0.5
1
1.5
2
2.5
D Cd
Depth/m
S01
S02
S03
S04
S05
S06
S07
S08
S09
S10

(F)

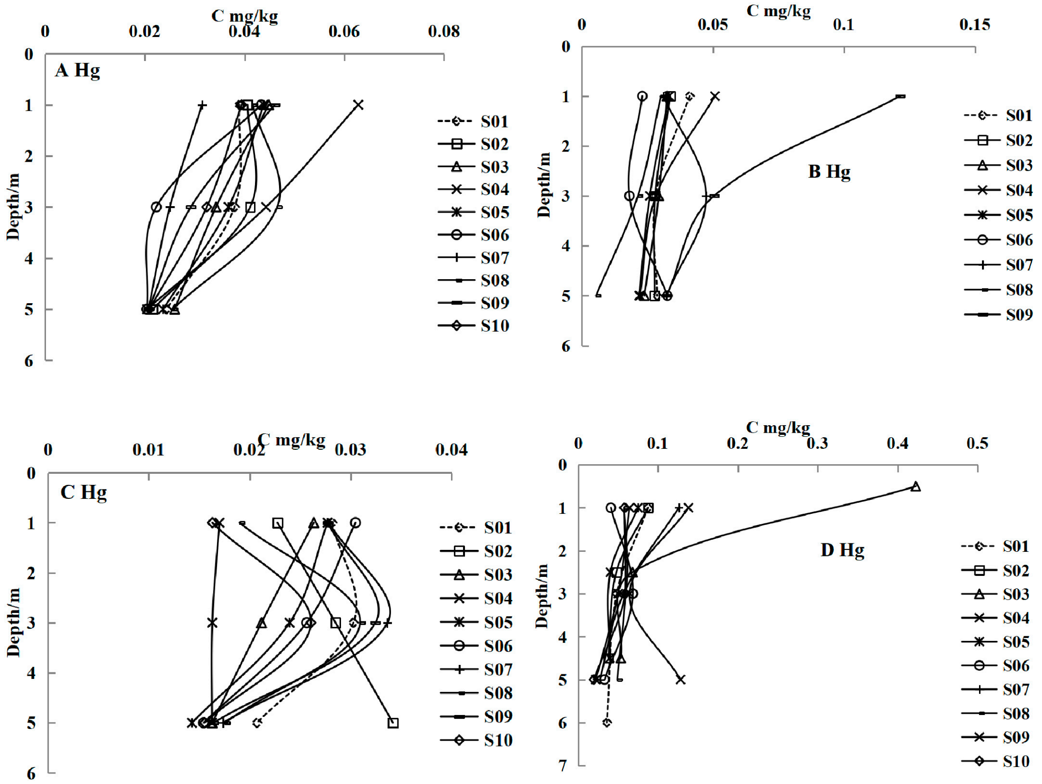

A Hg
C mg/kg
0 0.02 0.04 0.06 0.08
Depth/m
0 1 2 3 4 5 6
S01 S02 S03 S04 S05 S06 S07 S08 S09 S10
B Hg
C mg/kg
0 0.05 0.1 0.15
Depth/m
0 1 2 3 4 5 6
S01 S02 S03 S04 S05 S06 S07 S08 S09
C Hg
C mg/kg
0 0.01 0.02 0.03 0.04
Depth/m
0 1 2 3 4 5 6
S01 S02 S03 S04 S05 S06 S07 S08 S09 S10
D Hg
C mg/kg
0 0.1 0.2 0.3 0.4 0.5
Depth/m
0 1 2 3 4 5 6 7
S01 S02 S03 S04 S05 S06 S07 S08 S09 S10

(G)

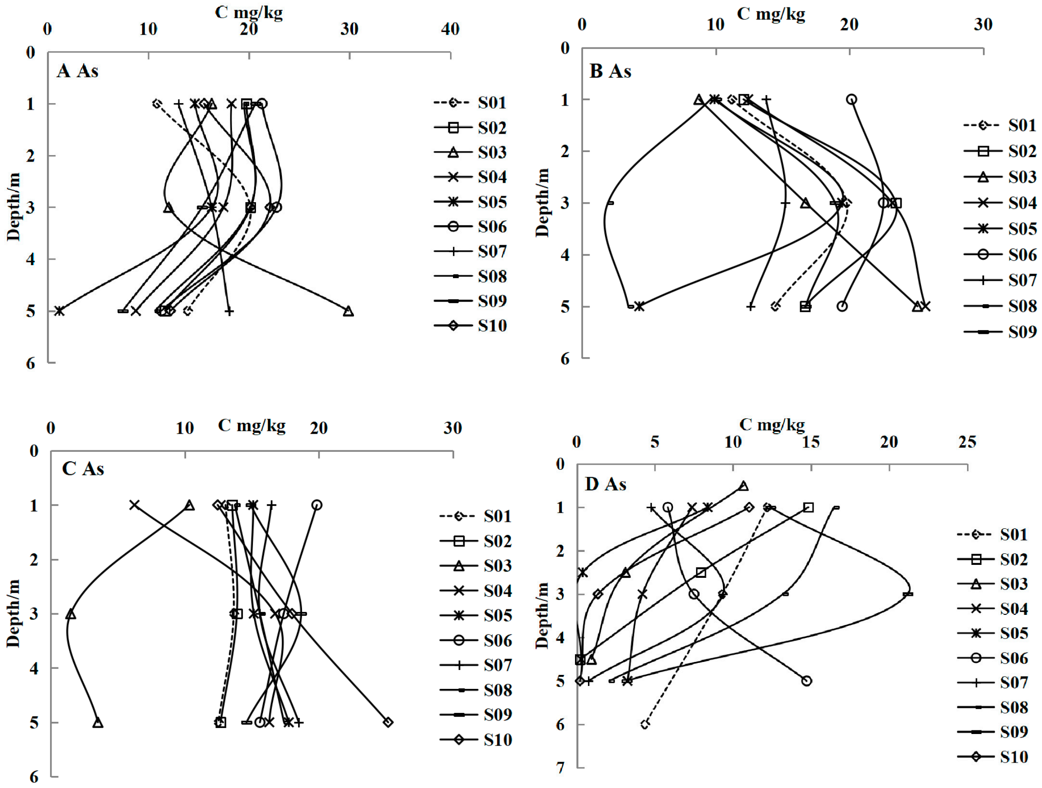

(H)

Figure S1. Vertical distribution of eight HM contents at different depths in different industrial plants.

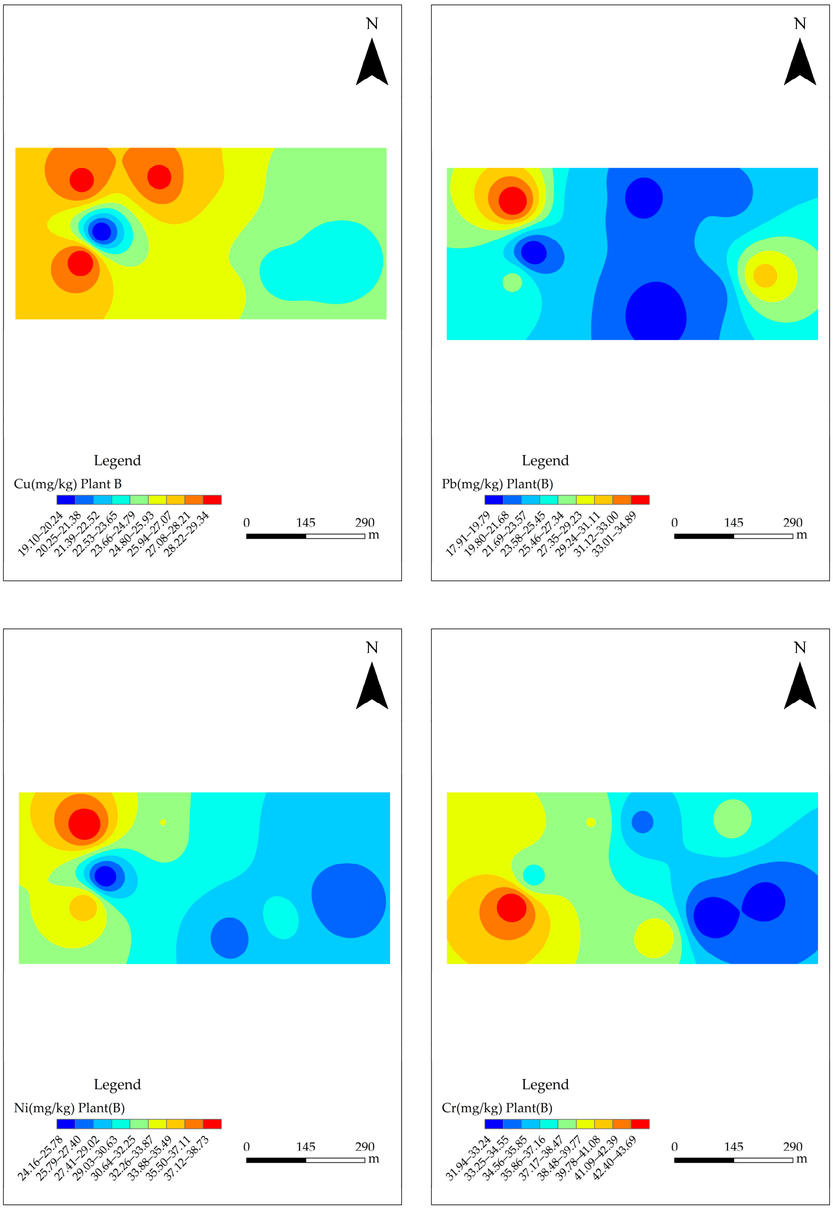

N
Legend
Cu(mg/kg) Plant B
19.10–20.24
20.25–21.38
21.39–22.52
22.53–23.65
23.66–24.79
24.80–25.93
25.94–27.07
27.08–28.21
28.22–29.34
0
145
290
m
N
Legend
Pb(mg/kg) Plant(B)
17.91–19.79
19.80–21.68
21.69–23.57
23.58–25.45
25.46–27.34
27.35–29.23
29.24–31.11
31.12–33.00
33.01–34.89
0
145
290
m
N
Legend
Ni(mg/kg) Plant(B)
24.16–25.78
25.79–27.40
27.41–29.02
29.03–30.63
30.64–32.25
32.26–33.87
33.88–35.49
35.50–37.11
37.12–38.73
0
145
290
m
N
Legend
Cr(mg/kg) Plant(B)
31.94–33.24
33.25–34.55
34.56–35.85
35.86–37.16
37.17–38.47
38.48–39.77
39.78–41.08
41.09–42.39
42.40–43.69
0
145
290
m

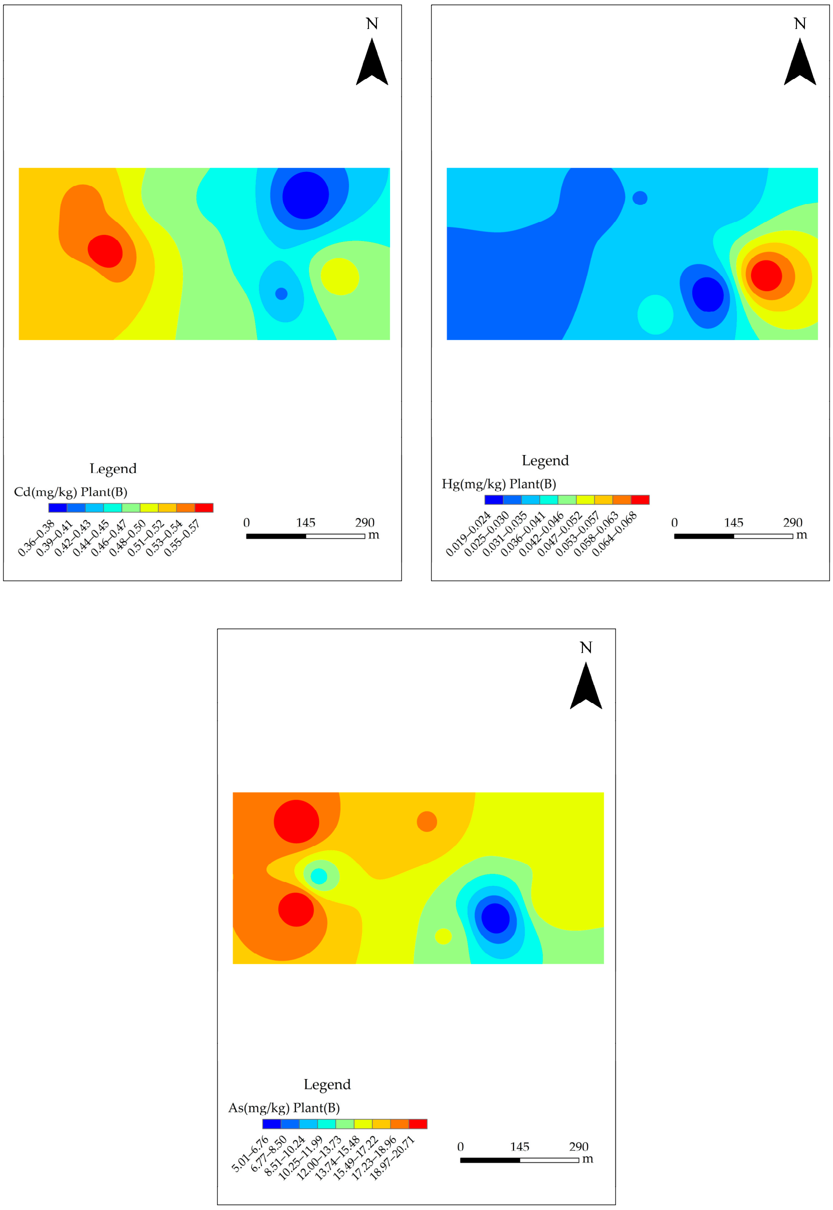
N
Legend
Cd(mg/kg) Plant(B)
0.36–0.38
0.39–0.41
0.42–0.43
0.44–0.45
0.46–0.47
0.48–0.50
0.51–0.52
0.53–0.54
0.55–0.57
0
145
290
m
N
Legend
Hg(mg/kg) Plant(B)
0.019–0.024
0.025–0.030
0.031–0.035
0.036–0.041
0.042–0.046
0.047–0.052
0.053–0.057
0.058–0.063
0.064–0.068
0
145
290
m
N
Legend
As(mg/kg) Plant(B)
5.01–6.76
6.77–8.50
8.51–10.24
10.25–11.99
12.00–13.73
13.74–15.48
15.49–17.22
17.23–18.96
18.97–20.71
0
145
290
m

(A)

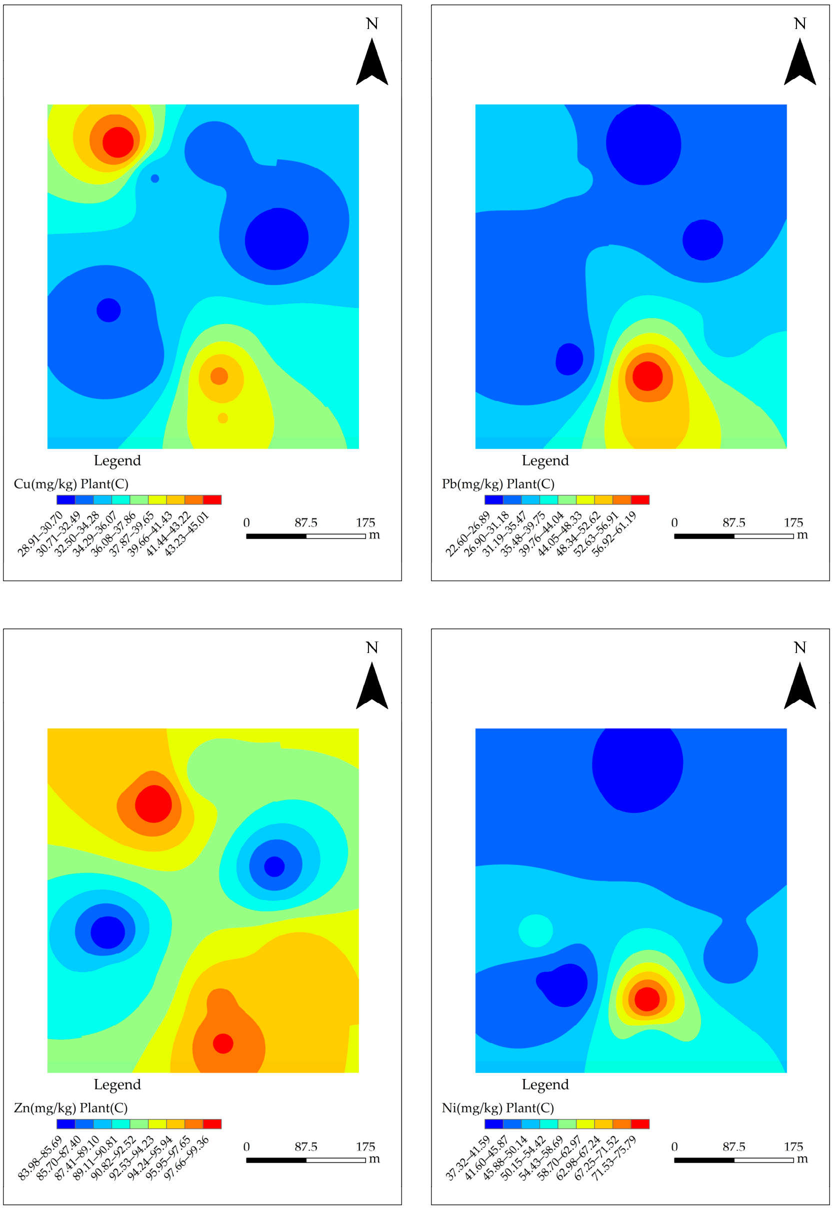
N
Legend
Cu(mg/kg) Plant(C)
28.91–30.70
30.71–32.49
32.50–34.28
34.29–36.07
36.08–37.86
37.87–39.65
39.66–41.43
41.44–43.22
43.23–45.01
0
87.5
175
m
N
Legend
Pb(mg/kg) Plant(C)
22.60–26.89
26.90–31.18
31.19–35.47
35.48–39.75
39.76–44.04
44.05–48.33
48.34–52.62
52.63–56.91
56.92–61.19
0
87.5
175
m
N
Legend
Zn(mg/kg) Plant(C)
83.98–85.69
85.70–87.40
87.41–89.10
89.11–90.81
90.82–92.52
92.53–94.23
94.24–95.94
95.95–97.65
97.66–99.36
0
87.5
175
m
N
Legend
Ni(mg/kg) Plant(C)
37.32–41.59
41.60–45.87
45.88–50.14
50.15–54.42
54.43–58.69
58.70–62.97
62.98–67.24
67.25–71.52
71.53–75.79
0
87.5
175
m

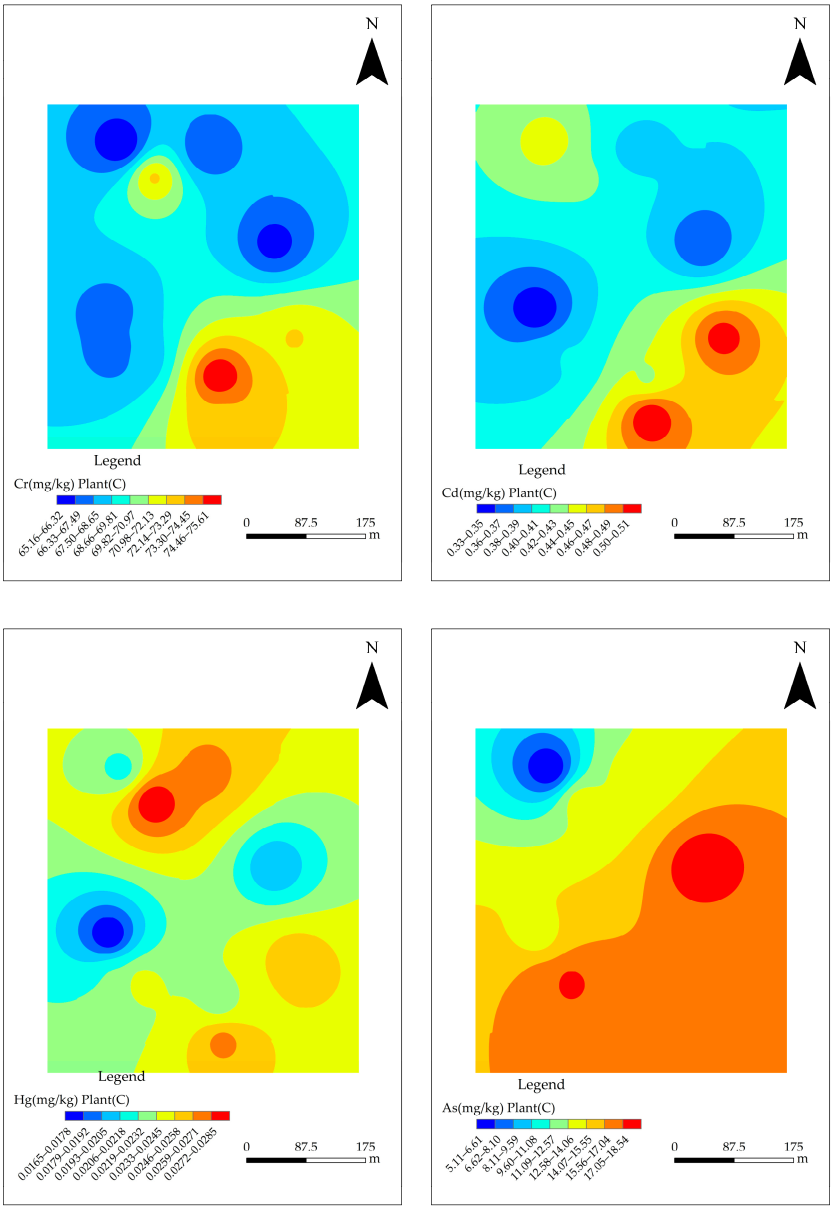
N
Legend
Cr(mg/kg) Plant(C)
65.16–66.32
66.33–67.49
67.50–68.65
68.66–69.81
69.82–70.97
70.98–72.13
72.14–73.29
73.30–74.45
74.46–75.61
0 87.5 175 m
N
Legend
Cd(mg/kg) Plant(C)
0.33–0.35
0.36–0.37
0.38–0.39
0.40–0.41
0.42–0.43
0.44–0.45
0.46–0.47
0.48–0.49
0.50–0.51
0 87.5 175 m
N
Legend
Hg(mg/kg) Plant(C)
0.0165–0.0178
0.0179–0.0192
0.0193–0.0205
0.0206–0.0218
0.0219–0.0232
0.0233–0.0245
0.0246–0.0258
0.0259–0.0271
0.0272–0.0285
0 87.5 175 m
N
Legend
As(mg/kg) Plant(C)
5.11–6.61
6.62–8.10
8.11–9.59
9.60–11.08
11.09–12.57
12.58–14.06
14.07–15.55
15.56–17.04
17.05–18.54
0 87.5 175 m

(B)

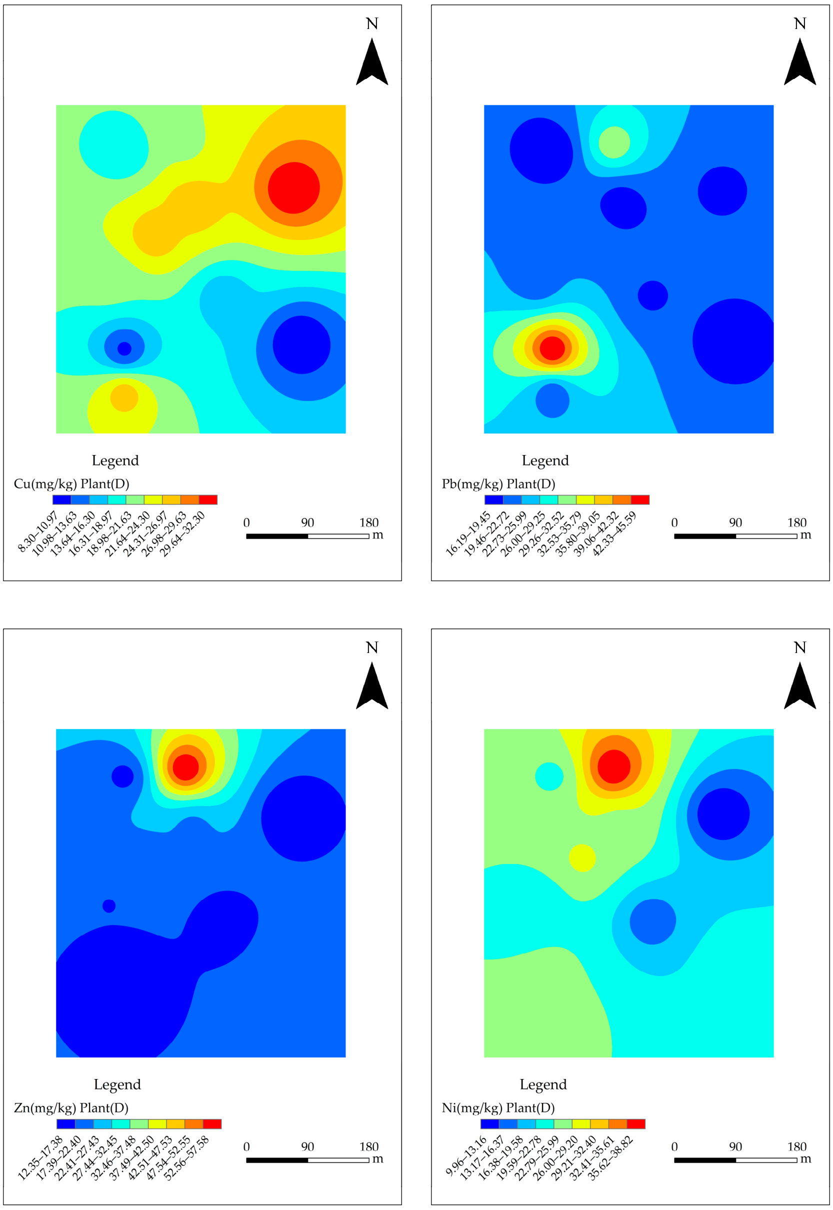
N
Legend
Cu(mg/kg) Plant(D)
8.30–10.97
10.98–13.63
13.64–16.30
16.31–18.97
18.98–21.63
21.64–24.30
24.31–26.97
26.98–29.63
29.64–32.30
0
90
180
m
N
Legend
Pb(mg/kg) Plant(D)
16.19–19.45
19.46–22.72
22.73–25.99
26.00–29.25
29.26–32.52
32.53–35.79
35.80–39.05
39.06–42.32
42.33–45.59
0
90
180
m
N
Legend
Zn(mg/kg) Plant(D)
12.35–17.38
17.39–22.40
22.41–27.43
27.44–32.45
32.46–37.48
37.49–42.50
42.51–47.53
47.54–52.55
52.56–57.58
0
90
180
m
N
Legend
Ni(mg/kg) Plant(D)
9.96–13.16
13.17–16.37
16.38–19.58
19.59–22.78
22.79–25.99
26.00–29.20
29.21–32.40
32.41–35.61
35.62–38.82
0
90
180
m

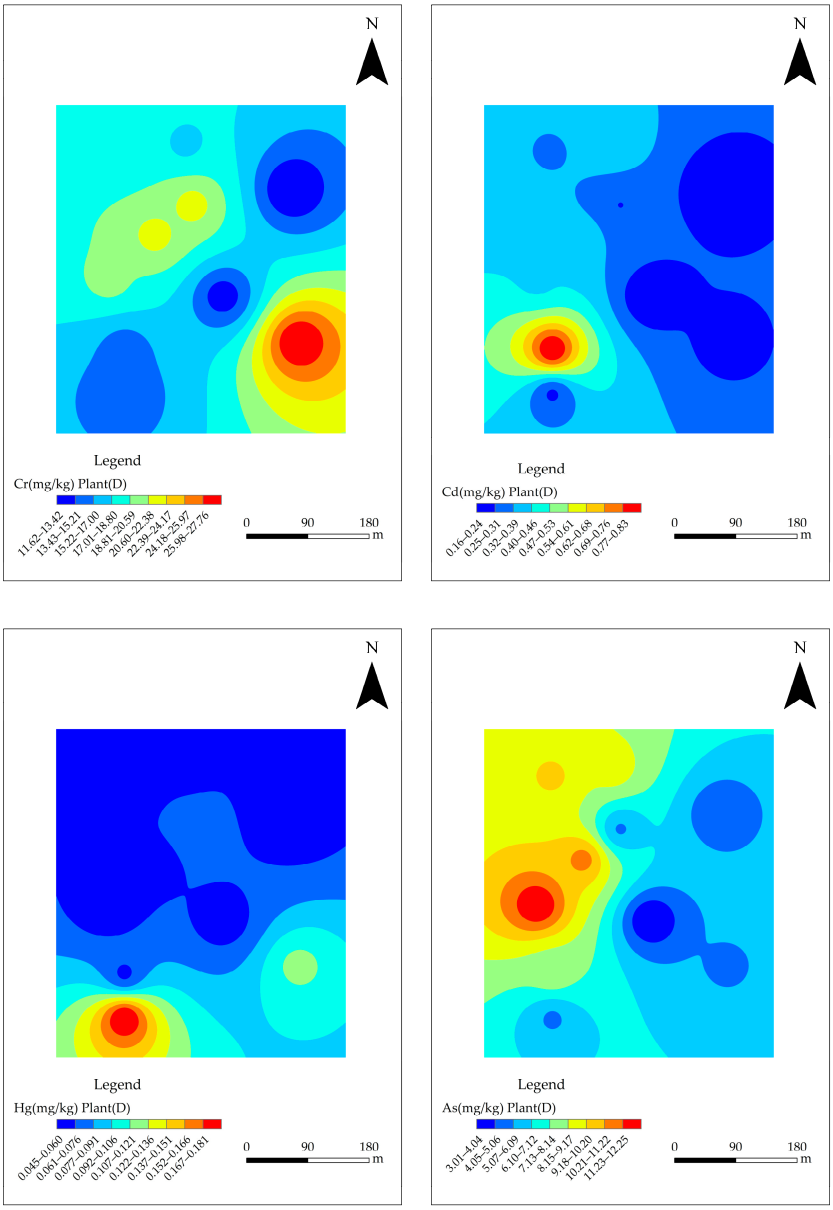

(C)

Figure S2. Spatial distribution characteristics of HMs in four industrial plants.

Table S1. The single factor contaminant index of HMs in different industrial plants.

	Index	Mean	Min	Max	SD	CV	Sum
	P_i-Cu	0.40	0.27	0.50	0.08	19.72	10
	P_i-Pb	0.35	0.23	0.55	0.10	29.87	10
	P_i-Zn	0.44	0.35	0.52	0.05	12.06	10
A	P_i-Ni	0.46	0.27	0.51	0.07	16.87	10
	P_i-Cr	0.41	0.31	0.46	0.05	12.15	10
	P_i-Cd	1.09	0.25	1.89	0.50	45.71	10
	P_i-Hg	0.02	0.01	0.03	0.003	18.46	10
	P_i-As	0.57	0.36	0.71	0.12	20.52	10
	P_i-Cu	0.23	0.19	0.29	0.04	16.20	9
	P_i-Pb	0.15	0.05	0.21	0.06	38.01	9
	P_i-Zn	--	--	--	--	--	--
B	P_i-Ni	0.25	0.21	0.34	0.04	17.01	9
	P_i-Cr	0.16	0.12	0.20	0.03	17.59	9
	P_i-Cd	1.23	0.91	1.48	0.20	16.42	9
	P_i-Hg	0.02	0.01	0.05	0.01	67.95	9
	P_i-As	0.40	0.29	0.67	0.11	28.72	9
	P_i-Cu	0.36	0.24	0.77	0.15	40.61	10
	P_i-Pb	0.20	0.14	0.27	0.04	20.25	10
	P_i-Zn	0.36	0.28	0.44	0.04	11.63	10
C	P_i-Ni	0.36	0.29	0.44	0.03	9.55	10
	P_i-Cr	0.32	0.26	0.36	0.03	8.30	10
	P_i-Cd	1.27	0.83	1.67	0.28	21.89	10
	P_i-Hg	0.01	0.007	0.013	0.002	21.28	10
	P_i-As	0.45	0.21	0.66	0.12	26.65	10
	P_i-Cu	0.24	0.09	0.65	0.15	62.78	10
	P_i-Pb	0.17	0.08	0.27	0.07	38.25	10
	P_i-Zn	0.11	0.04	0.21	0.05	45.90	10
D	P_i-Ni	0.28	0.08	0.58	0.17	60.02	10
	P_i-Cr	0.13	0.06	0.34	0.08	62.30	10
	P_i-Cd	0.88	0.00	1.94	0.57	65.51	10
	P_i-Hg	0.05	0.02	0.18	0.04	96.94	10
	P_i-As	0.35	0.16	0.55	0.13	36.62	10
	P_i-Cu	0.31	0.09	0.77	0.13	43.19	39
	P_i-Pb	0.21	0.05	0.55	0.11	47.92	39
	P_i-Zn	0.30	0.04	0.52	0.15	50.28	39
Average	P_i-Ni	0.34	0.08	0.58	0.12	35.92	39
	P_i-Cr	0.26	0.06	0.46	0.13	50.01	39
	P_i-Cd	1.11	0.00	1.94	0.43	38.96	39
	P_i-Hg	0.02	0.007	0.18	0.03	116.83	39
	P_i-As	0.44	0.16	0.71	0.14	32.08	39

Table S2. HQs and HIs of soil HMs under different exposure pathways in different industrial plants.

			A				B		
		HQ_{ois}	HQ_{dcs}	HQ_{pis}	HI	HQ_{ois}	HQ_{dcs}	HQ_{pis}	HI
Cu	Mean	6.05E-03	3.45E-05	-	6.08E-03	3.47E-03	1.98E-05	-	3.49E-03
Zn	Mean	2.23E-03	1.27E-05	-	2.24E-03	-	-	-	-
Hg	Mean	8.70E-04	7.10E-05	4.03E-05	9.81E-04	8.87E-04	7.23E-05	4.11E-05	1.00E-03
Cd	Mean	1.97E-03	4.49E-04	9.13E-03	1.15E-02	2.22E-03	5.07E-04	1.03E-02	1.30E-02
Ni	Mean	1.35E-02	1.93E-03	1.39E-01	1.54E-01	7.57E-03	1.08E-03	7.79E-02	8.66E-02
Cr	Mean	1.65E-01	3.78E-02	2.30E-01	4.33E-01	6.25E-02	1.43E-02	8.70E-02	1.64E-01
As	Mean	3.41E-01	1.95E-03	3.16E-01	6.59E-01	2.41E-01	1.38E-03	2.23E-01	4.66E-01
			C				D		
		HQ_{ois}	HQ_{dcs}	HQ_{pis}	HI	HQ_{ois}	HQ_{dcs}	HQ_{pis}	HI
Cu	Mean	5.50E-03	3.14E-05	-	5.54E-03	3.68E-03	2.10E-05	-	3.69E-03
Zn	Mean	1.81E-03	1.04E-05	-	1.82E-03	5.40E-04	3.08E-06	-	5.43E-04
Hg	Mean	4.89E-04	3.99E-05	2.27E-05	5.52E-04	2.32E-03	1.89E-04	1.08E-04	2.62E-03
Cd	Mean	2.29E-03	5.23E-04	1.06E-02	2.68E-02	1.58E-03	3.62E-04	7.34E-03	9.28E-03
Ni	Mean	1.09E-02	1.56E-03	1.12E-01	1.25E-01	8.43E-03	1.20E-03	8.69E-02	9.65E-02
Cr	Mean	1.29E-01	2.94E-02	1.79E-01	3.37E-01	5.23E-02	1.19E-02	7.25E-02	1.37E-01
As	Mean	2.73E-01	1.56E-03	2.53E-01	5.27E-01	2.09E-01	1.19E-03	1.94E-01	4.03E-01
	HQ_{ois}	HQ_{dcs}	HQ_{pis}	HI					
Cu	4.70E-03	2.69E-05	-	4.73E-03					
Zn	1.53E-03	8.72E-06	-	1.54E-03					
Hg	1.15E-03	9.37E-05	5.32E-05	1.30E-03					
Cd	2.01E-03	4.59E-04	9.32E-03	1.18E-02					
Ni	1.02E-02	1.45E-03	1.05E-01	1.16E-01					
Cr	1.03E-01	2.36E-02	1.43E-01	2.70E-01					
As	2.67E-01	1.52E-03	2.47E-01	5.15E-01					
